# The prenatal challenge with lipopolysaccharide and polyinosinic:polycytidylic acid disrupts CX3CL1-CX3CR1 and CD200-CD200R signalling in the brains of male rat offspring: a link to schizophrenia-like behaviours

**DOI:** 10.1186/s12974-020-01923-0

**Published:** 2020-08-23

**Authors:** Katarzyna Chamera, Katarzyna Kotarska, Magdalena Szuster-Głuszczak, Ewa Trojan, Alicja Skórkowska, Bartosz Pomierny, Weronika Krzyżanowska, Natalia Bryniarska, Agnieszka Basta-Kaim

**Affiliations:** 1 Laboratory of Immunoendocrinology, Department of Experimental Neuroendocrinology, Maj Institute of Pharmacology, Polish Academy of Sciences, 12 Smętna St, 31-343 Kraków, Poland; 2 Department of Toxicology, Faculty of Pharmacy, Jagiellonian University Collegium Medicum, 9 Medyczna St, 30-688 Kraków, Poland

**Keywords:** Maternal immune activation, Lipopolysaccharide, Polyinosinic:polycytidylic acid, CX3CL1-CX3CR1, CD200-CD200R, Microglia, Schizophrenia

## Abstract

**Background:**

The bidirectional communication between neurons and microglia is fundamental for the homeostasis and biological function of the central nervous system. Maternal immune activation (MIA) is considered to be one of the factors affecting these interactions. Accordingly, MIA has been suggested to be involved in several neuropsychiatric diseases, including schizophrenia. The crucial regulatory systems for neuron-microglia crosstalk are the CX3CL1-CX3CR1 and CD200-CD200R axes.

**Methods:**

We aimed to clarify the impact of MIA on CX3CL1-CX3CR1 and CD200-CD200R signalling pathways in the brains of male Wistar rats in early and adult life by employing two neurodevelopmental models of schizophrenia based on the prenatal challenge with lipopolysaccharide (LPS) and polyinosinic:polycytidylic acid (Poly I:C). We also examined the effect of MIA on the expression of microglial markers and the profile of cytokines released in the brains of young offspring, as well as the behaviour of adult animals. Moreover, we visualized the localization of ligand-receptor systems in the hippocampal regions (CA1, CA3 and DG) and the frontal cortex of young rats exposed to MIA. The differences between groups were analysed using Student’s *t* test.

**Results:**

We observed that MIA altered developmental trajectories in neuron-microglia communication in the brains of young offspring, as evidenced by the disruption of CX3CL1-CX3CR1 and/or CD200-CD200R axes. Our data demonstrated the presence of abnormalities after LPS-induced MIA in levels of *Cd40*, *Il-1β*, *Tnf-α*, *Arg1*, *Tgf-β* and *Il-10*, as well as IBA1, IL-1β and IL-4, while after Poly I:C-generated MIA in levels of *Cd40*, *iNos*, *Il-6*, *Tgf-β*, *Il-10*, and IBA1, IL-1β, TNF-α, IL-6, TGF-β and IL-4 early in the life of male animals. In adult male rats that experienced prenatal exposure to MIA, we observed behavioural changes resembling a schizophrenia-like phenotype.

**Conclusions:**

Our study provides evidence that altered CX3CL1-CX3CR1 and/or CD200-CD200R pathways, emerging after prenatal immune challenge with LPS and Poly I:C, might be involved in the aetiology of schizophrenia.

## Background

Bidirectional communication between neurons and microglia is fundamental for the homeostasis and biological function of the central nervous system (CNS). This crosstalk orchestrates the balance for proper neurodevelopmental processes, including neurogenesis, synaptogenesis, synaptic pruning, axonal growth, astrocyte maturation, mitochondrial biogenesis, myelination and blood-brain barrier integrity [1, 2]. This communication is also crucial for the control of the immune response [3–5]. In the brain, microglia are the major immunocompetent cells involved in the induction and resolution of inflammatory processes [6, 7]. However, to maintain microglia in a resting phenotype, the exchange of signals linking microglia with neurons by various endogenous systems is required [8, 9]. The dysfunction of this dynamic crosstalk leads to microglial activation, which is manifested by increased phagocytic activity, mobility and the production of pro-inflammatory factors [10].

In this context, CX3CL1-CX3CR1 and CD200-CD200R interactions are crucial and represent unique ligand-receptor axes. CX3CL1 (fractalkine) is the only member of the CX3C chemokine family [11], and it exhibits remarkably higher expression in the brain than in the periphery, indicating a unique role of this ligand in the CNS [12]. CX3CL1 originates mostly from neurons, while its only known corresponding receptor, CX3CR1, is present on microglial cells [13–15]. In addition to the induction of chemotaxis, the CX3CL1-CX3CR1 axis regulates neurodevelopmental processes, including neuronal survival [16, 17] and synaptic pruning [18] as well as the reactivity of microglia and inflammatory cytokine release [19, 20]. Another important inhibitory signalling dyad in the brain is the CD200-CD200R axis. CD200 (known also as OX-2) is a membrane glycoprotein expressed ubiquitously on neurons, endothelial cells and oligodendrocytes, and it plays a critical role in regulating and maintaining the resting state of microglial cells [21]. The cognate receptor of CD200 (CD200R) is present almost exclusively in myeloid cells, including microglia [21–23]. Data have shown that disturbances in CD200-CD200R signalling potentiate the pro-inflammatory response of microglia to immune stimuli [24] and lead to a prolonged inflammatory response as well as to neurodegeneration [3, 25]. Moreover, the malfunction of the CD200-CD200R axis has been observed in ageing [26].

Numerous studies in different experimental settings demonstrate a direct association between neurodevelopmental malfunctions in the brain immune system and the occurrence of schizophrenia in adulthood [27]. A significant link between maternal immune activation (MIA) and increased risk of this disease in the offspring has been demonstrated in a variety of retrospective epidemiological studies [28, 29]. Notably, exposure to MIA is capable of enhancing the pro-inflammatory response in the three maternal-foetal compartments, namely, the placenta, the amniotic fluid and the foetus, including the foetal brain [30, 31]. The sensitivity of microglia to MIA and their engagement in many developmental processes make them an attractive candidate for orchestrating neurodevelopment mediated by the CX3CL1-CX3CR1 and/or CD200-CD200R systems.

Therefore, in the present study, we sought to verify the hypothesis that MIA affects CX3CL1-CX3CR1 and/or CD200-CD200R signalling in early life, which is the period when crucial neurodevelopmental processes occur. For this purpose, we performed studies in two commonly accepted neurodevelopmental models of schizophrenia that are based on prenatal challenge with lipopolysaccharide (LPS) [32, 33] and polyinosinic:polycytidylic acid (Poly I:C) [31, 34]. MIA induced by LPS treatment mimics infection with Gram-negative bacteria [35], while that elicited by Poly I:C is similar to the acute phase response to viral infection [36]. In the offspring at postnatal day 7 (PND7), we examined the impact of MIA on the mRNA and protein expression of CX3CL1, CD200 and their receptors in the hippocampus and frontal cortex, which are areas of the brain distinctly affected in schizophrenia [37, 38]. Moreover, we visualized the localization of ligand-receptor systems in hippocampal regions (CA1, CA3 and DG) and the frontal cortex after MIA. Considering that CX3CL1-CX3CR1 and CD200-CD200R interactions are crucial for the modulation of microglial reactivity, we explored the expression of microglial markers and the profile of cytokines released in the brains of offspring at PND7. Next, for further characterization of the prenatal LPS and Poly I:C immune challenge, we assessed not only the CX3CL1-CX3CR1 and CD200-CD200R systems at PND93 but also the behavioural status of adult male rat offspring (at PND30 and PND88-92).

## Materials and methods

### Animals

Adult Wistar rats (females 226–250 g and males 251–275 g upon arrival) were purchased from Charles River (Sulzfeld, Germany). The animals were maintained under standard conditions: room temperature of 23 °C, 12/12-h light/dark cycle, lights on at 6:00 am, and ad libitum access to water and food. The phase of the oestrous cycle was determined based on vaginal smears that were obtained daily from the females. On the day of pro-oestrus, the females were placed with males for 12 h, and the presence of sperm in vaginal smears was checked the next morning [defined as gestational day 1 (GD1)]. Pregnant females (*n* = 28) were randomly divided into four equal groups: (1) control for LPS (kLPS), (2) control for Poly I:C (kPoly), (3) LPS and (4) Poly I:C. All procedures were approved by the Animal Care Committee of Maj Institute of Pharmacology, Polish Academy of Sciences, Cracow, and met the criteria of the International Council for Laboratory Animals and Guide for the Care and Use of Laboratory Animals (consent number: 236/2016). All possible efforts were made to minimize the number of animals used and their suffering.

### Prenatal treatment with LPS and Poly I:C

**Fig. 1:**
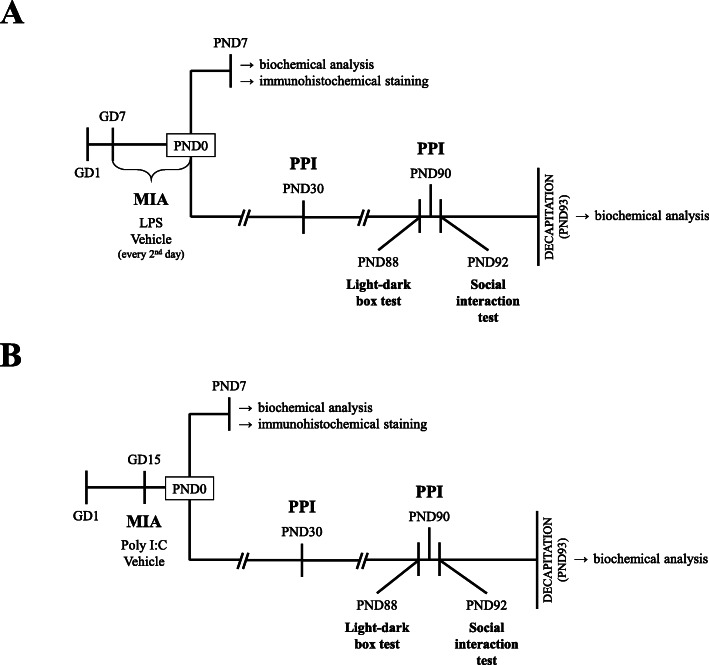
Experimental design. Pregnant dams were exposed to MIA with either LPS (2 mg/kg in 1 ml) beginning at GD7 and continuing every second day until delivery (**a**) or a single injection of Poly I:C (4 mg/kg in 1 ml) at GD15 (**b**). Control groups were subjected to vehicle (saline) injections in a corresponding manner. At PND7, some male offspring were sacrificed and used for biochemical and immunohistofluorescent analyses. The rest of the animals underwent the behavioural examination, including the PPI test at PND30 and PND90, the light-dark box test at PND88 and the social interaction test at PN92. Twenty-four hours after the last behavioural test (PND93), the rats were sacrificed by decapitation and the tissues were collected for biochemical examination

LPS (from *Escherichia coli* 026:B6, Sigma-Aldrich, St. Louis, MO, USA) was dissolved in saline to produce a 2-mg/kg solution in 1 ml, and it was administrated subcutaneously to pregnant rats beginning on GD7 and then again every second day until delivery [32]. The kLPS group received an appropriate amount of vehicle (saline). Poly I:C was purchased from Sigma-Aldrich (St. Louis, MO, USA) as a sodium salt and was dissolved in saline to obtain 1 ml of a 4-mg/kg solution. Poly I:C was administered via the tail vein of pregnant rats of the Poly I:C group on GD15 [39], while the kPoly group received an appropriate injection of vehicle (saline). No differences in litter size and weight were observed between groups. Only male offspring were used in the present study due to the consent (number: 236/2016) of the Animal Care Committee. The females were included in another research not presented within this article. At PND7, some of the male offspring were sacrificed and used for biochemical and immunohistochemical analyses. At PND21, the rest of the offspring were separated from dams and housed in groups of five per cage under standard conditions. Those rats were left for behavioural (at PND30 and PND88-92) and biochemical examinations (PND93) (please refer to Fig. 1). The behavioural experiments were performed between 9:00 am and 12:00 am. The investigators were not blinded to the experimental conditions. The numbers of animals included in each analysis are presented in the description of the method and the caption to the corresponding figure or table.

### Biochemical study

#### Tissue collection and fixation

At PND7, the male offspring from the experimental groups (LPS and Poly I:C) and the control groups (kLPS and kPoly) were sacrificed under non-stress conditions. For the immunohistofluorescent analyses, whole brains were isolated, immediately immersed in a solution of 4% paraformaldehyde in PBS (Santa Cruz Biotechnology, Dallas, TX, USA) and were incubated overnight at 4 °C. Next, tissues were cut into coronal 20-μm sections using an automatic cryostat Leica CM1860 (Leica, Wetzlar, Germany) and placed on microscopic slides. Until staining, the slides were stored at − 20 °C. For biochemical analyses, the hippocampi and the frontal cortices of male offspring at PND7 and PND93 were dissected on an ice-cold glass plate, and then they were stored at − 80 °C before being used for further treatment.

#### Quantitative real-time polymerase chain reaction (qRT-PCR)

Total RNA was extracted from the hippocampi and frontal cortices of PND7 offspring using a GeneMATRIX Universal RNA Purification Kit (EURx, Gdańsk, Poland) according to the manufacturer’s instructions. Immediately after extraction, the concentration of RNA was determined by a NanoDrop Spectrophotometer (ND/1000 UV/Vis, Thermo Fisher NanoDrop, Waltham, MA, USA). The synthesis of complementary DNA (cDNA) was performed via reverse transcription from equal amounts of RNA (1 μg) using an NG dART RT kit (EURx, Gdańsk, Poland). The cDNA was amplified with a FastStart Universal Probe Master (Rox) kit (Roche, Basel, Switzerland) and TaqMan probes (Thermo Fisher Scientific, Waltham, MA, USA) for the following genes: *Cx3cl1*, *Cx3cr1*, *Cd200*, *Cd200r*, *MhcII*, *Cd68*, *Cd40*, *iNos*, *Il-1β*, *Tnf-α*, *Il-6*, *Arg1*, *Igf-1*, *Tgf-β*, *Il-4* and *Il-10* (corresponding catalogue numbers of TaqMan probes are presented in Supplementary Table 1). *B2m* or *Hprt* served as an internal control for sample normalization. The PCR products were generated in mixtures consisting of cDNA, which was used as the PCR template (1 μl), TaqMan forward and reverse primers (1 μl), 1× FastStart Universal Probe Master (Rox) mix, containing 250-nM hydrolysis probe labelled with the fluorescent reporter dye [fluorescein (FAM)] at the 5′-end and a quenching dye at the 3′-end (10 μl), and finally the remainder of PCR-grade distilled water to reach a total volume of 20 μl. Thermocycling conditions contained an initial denaturation at 95 °C for 10 min, followed by 40 cycles of denaturation at 95 °C for 15 s, annealing at 60 °C for 1 min and extension at 50 °C for 2 min. The threshold value (*C*_*t*_) for each sample was set in the exponential phase of PCR, and the ∆∆*C*_*t*_ method was used for data analysis with *n* = 4–8 in each group.

#### Tissue preparation and determination of protein concentration

The collected tissues were homogenized by a Tissue Lyser II (Qiagen Inc, Valencia, CA, USA) in RIPA lysis buffer containing protease inhibitor cocktail, phosphatase inhibitor cocktail, 1 mM sodium orthovanadate and 1 mM phenylmethanesulfonyl fluoride (all reagents from Sigma-Aldrich, St. Louis, MO, USA). The protein concentrations in the analysed samples were determined using a BCA Protein Assay Kit (Sigma-Aldrich, St. Louis, MO, USA) with bovine serum albumin as a standard, and measurements were collected at a wavelength of 562 nm using a Tecan Infinite 200 Pro spectrophotometer (Tecan, Mannedorf, Germany). The prepared samples were stored at − 20 °C before being used to examine the required biochemical parameters.

#### Enzyme-linked immunosorbent assay (ELISA)

The protein levels of CX3CL1 (Cloud-Clone Corp., Katy, TX, USA); CX3CR1, CD200, CD200R, IL-1β, IL-4, IL-10, IBA1 (all from Cusabio, Houston, TX, USA); IL-6, TGF-β (Fine Test, Wuhan, Hubei, China); and TNF-α (Thermo Fisher Scientific, Waltham, MA, USA) in the hippocampi and frontal cortices (*n* = 6–8 in each group) of male rats at PND7 were measured using commercially available ELISA kits. The procedures were performed in accordance with the manufacturer’s instructions, and the minimum detectable doses were CX3CL1 0.055 ng/ml, CX3CR1 7.8 pg/ml, CD200 11.75 pg/ml, CD200R 4.67 pg/ml, IL-1β 15.6 pg/ml, TNF-α 16 pg/ml, IL-6 37.5 pg/ml, TGF-β 18.75 pg/ml, IL-4 3.9 pg/ml, IL-10 0.78 pg/ml and IBA1 6.25 pg/ml. Intra- and inter-assay precision values were CX3CL1 < 10%, <12%, and CX3CR1, CD200, CD200R, IL-1β, TNF-α, IL-6, TGF-β, IL-4, IL-10 and IBA1 < 8%, < 10%, respectively. The protein levels of CX3CL1, CX3CR1, CD200, CD200R and IBA1 were also assessed in the hippocampi and frontal cortices (*n* = 6–9 in each group) of adult male offspring (PND93).

#### Immunohistofluorescent staining

Antigen retrieval was carried out using trisodium citrate buffer solution (pH ≈ 9) (Sigma-Aldrich, St. Louis, MO, USA) in glass containers, which were placed in a water bath for 30 min at 80 °C. The containers were removed from the water bath and allowed to return to room temperature. Sections were washed twice in 0.2% Tween in PBS solution (PBST). For nonspecific antibody binding inhibition, sections were blocked using 10% donkey normal serum (DNS) (Abcam, Cambridge, UK) or 10% donkey and goat serum (1:1) (DNS+GNS) (Abcam, Cambridge, UK) solution in PBST for 1 h at room temperature. The type of serum solution was determined by the combination of secondary antibodies used for staining. After blocking, the medium was removed, and sections were immersed in appropriate primary antibody solutions (anti-CX3CL1: ab25088, 1:100; anti-CX3CR1: ab8021, 1:100; anti-OX2: ab203887, 1:100, all from Abcam, Cambridge, UK, and anti-OX2R: AOR-002, 1:50, Alomone labs, Jerusalem, Israel). Proteins were co-stained with neuronal (anti-MAP2: 5392, 1:1000, Abcam, Cambridge, UK), astroglial (anti-GFAP: ab4674, 1:1000, Abcam, Cambridge, UK) or microglial (anti-IBA1: ab5076, 1:200, Abcam, Cambridge, UK) markers. Each mixture of two specific antibodies was dissolved in an appropriate 2% DNS or DNS+GNS serum in PBST. Tissues were incubated with a mixture of two specific primary antibodies solutions at 4 °C overnight. Next, slides were washed twice in 2% DNS or DNS+GNS in PBST. Sections were incubated in an appropriate mixture of two secondary antibodies (goat anti-chicken Alexa Fluor 488: A11039, 1:300, Thermo Fisher Scientific, Waltham, MA, USA; donkey anti-mouse TR: sc2785, 1:300, donkey anti-goat FITC: sc2024, 1:300, Santa Cruz Biotechnology, Dallas, TX, USA; and donkey anti-rabbit TR: ab6800, 1:1000, Abcam, Cambridge, UK), which were in a PBST solution, and then they were incubated for 1 h in the dark at room temperature. Tissues were washed three times in PBST, dried, mounted in Fluoroshield mounting medium (Sigma-Aldrich, St. Louis, MO, USA) and cover-slipped. Visualization of staining was performed using a Leica DMI8 fluorescence inverted microscope with the objective HCX FLUOTAR semi-plan apochromatic (a total magnification × 400) (both from Leica, Wetzlar, Germany), with magnification applied for all images × 40. Images of the DG (precisely, the polymorph layer dentate gyrus), CA1 (the images covered the oriens layer hippocampus, pyramidal cell layer hippocampus and radiatum layer hippocampus) and CA3 fields of the hippocampus and frontal cortex (precisely, dorsolateral entorhinal cortex) were captured using a Leica DFC450 digital CCD camera (Leica, Wetzlar, Germany). At least two fields of the structures per section were visualized, and two sections per animal (*n* = 2 in each group) per staining were used to generate representative images. Immunohistofluorescent staining was performed only to visualize the localization of the receptors and ligands on appropriate cell types.

### Behavioural study

#### Prepulse inhibition test (PPI)

At PND30 and PND90, the male offspring from the experimental groups (LPS and Poly I:C) and the control groups (kLPS and kPoly) underwent the PPI. The PPI procedure was adopted with some modifications from our previously published studies [32, 40, 41]. PPI was tested in eight ventilated startle chambers (SR-LAB, San Diego Instruments, California, USA) with a single Plexiglas cylinder (inner diameter of 9 cm) mounted in each of them. A high-frequency loudspeaker inside each chamber produced both continuous background noise of 65 dB and various acoustic stimuli. The average startle amplitudes (AVGs) were detected for each animal by a piezoelectric accelerometer, and then the data were digitized and used for subsequent analyses. Before the experiments, each chamber was individually calibrated by the external sensor to display a similar readout of the reference stimulus. The AVGs were measured during the 200-ms recording window. After 5 min of habituation with the background noise, four types of acoustic stimuli were used in random order. Each trial consisted of either a single pulse alone [intensity 120 dB, duration 40 ms, (P)] or a pulse preceded by a prepulse at one out of three intensities [70, 75 and 80 dB; duration 20 ms; (PP)] applied 80 ms before a pulse. During each experimental session, 20 trials of each type were presented with an interstimulus interval of 20 s. The AVGs were recorded, and the percentage of PPI (%PPI) induced by each prepulse intensity was calculated as %PPI = [(P − PP)/P] × 100%. The number of animals used in the PPI test was as follows: *n* = 23 in the kLPS and LPS groups, *n* = 21 in the kPoly group and *n* = 14 in the Poly I:C group.

#### Light-dark box test

The light-dark box test was performed using an apparatus consisting of four cages with a computer-controlled system (TSE Systems, Bad Homburg, Germany) based on the procedure reported by Chocyk et al. [42]. Each experimental box had two compartments: light (covering ¾ of the cage, brightly lit – 100 lx) and dark (covered with a lid), which were made of clear and black acrylic, respectively. Both sections were permeable to infrared light and were connected by a central gate (10.6 cm × 10.4 cm). Therefore, the two parts of the cage were freely accessible for the animals to explore. The experimental boxes were located in soundproof, ventilated cabinets on base constructions that contained integrated infrared sensors along the horizontal and vertical axes. One hour before the test, male rats (PND88) were kept in total darkness. The entire experiment was also conducted in a dark room. At the beginning of each testing session, which lasted 10 min, an animal was placed in one corner of the light compartment, facing away from the gate. The behavioural response during the trials was recorded by Fear Conditioning Software (TSE, Bad Homburg, Germany). Specifically, the time spent in each compartment, the distance travelled and the average speed were calculated for each animal (*n* = 8–9 in the kLPS and LPS groups, *n* = 19–21 in the kPoly and Poly I:C groups).

#### Social interaction test

The experiments investigating social interactions of the animals (*n* = 6 in each group) were conducted based on a protocol described by Bator et al. [43] in an open field space (60 × 60 × 30 cm) made of black Plexiglas and dimly illuminated (18 lx) with indirect light. The day before the test, male offspring (PND91) were transferred to the experimental room and were allowed to individually adapt to the open field arena for 7 min. Afterwards, half of the rats were marked with potassium permanganate on the rear part of their bodies. On the test day (PND92), two unfamiliar animals (one unmarked and one marked) that received identical prenatal treatment were placed in the open field arena. The behaviour of the rats was observed for 10 min by two independent experimenters. The following social behaviours were scored: (1) non-aggressive consisting of following (rat’s movement towards and following the other rat), sniffing (sniffing parts of the other rat’s body, including an anogenital region) and social grooming (licking and chewing a fur of the other animal), and (2) aggressive consisting of attacking, fighting and aggressive grooming (aggressive licking and chewing a fur of another rat). During the test, the time and number of all types of events were measured for each separate animal. Social interactions were expressed as summed scores of the time and the number of aggressive and non-aggressive activities.

### Statistical data analysis

Statistical analysis of the data was performed using Statistica 13.0 Software (StatSoft, Palo Alto, CA, USA). The results from qRT-PCR studies are displayed as average fold change ± standard errors of the mean (SEMs). The results of ELISA experiments are presented as the means ± SEMs. The data from behavioural examinations are demonstrated as the means ± SEMs. The normal distribution and the homogeneity of the variance were examined using the Shapiro-Wilk test and Levene’s test, respectively. Comparisons of variables between groups (kLPS vs. LPS or kPoly vs. Poly I:C) were analysed using Student’s *t* test. The results were considered statistically significant when the *p* value was lower than 0.05. All graphs were prepared with GraphPad Prism 7 software (San Diego, CA, USA).

## Results

### The impact of MIA generated by LPS and Poly I:C treatment on *Cx3cl1*, *Cx3cr1*, *Cd200* and *Cd200r* expression in the hippocampi and the frontal cortices of offspring at PND7

In the first set of experiments, to analyse the influence of MIA induced by LPS and Poly I:C treatment on the mRNA expression of neuronal ligands and their corresponding microglial receptors, we determined *Cx3cl1*, *Cx3cr1*, *Cd200* and *Cd200r* levels by qRT-PCR in the hippocampi and the frontal cortices of male offspring at PND7 (Table 1). Student’s *t* test analysis showed a significant increase in *Cx3cl1* (*p* = 0.0316) and *Cd200* (*p* = 0.0344) expression in the hippocampus of the prenatally LPS-treated offspring compared to that of the control group. The hippocampal levels of *Cx3cr1* and *Cd200r* were not affected by MIA with LPS, and there were no changes observed in the frontal cortex of the animals from the LPS group in comparison to the kLPS group. In contrast, the prenatally Poly I:C-exposed young rats displayed only a decrease in *Cd200* (*p* = 0.0145) mRNA expression in the frontal cortex when compared with that of the kPoly group. The examination of *Cx3cl1*, *Cx3cr1* and *Cd200r* expression in both brain areas, but also *Cd200* in the hippocampus of the Poly I:C group revealed no significant differences in mRNA levels.

**Table 1:** The effect of MIA induced by LPS and Poly I:C treatment on the gene expression of *Cx3cl1*, *Cx3cr1*, *Cd200* and *Cd200r* in the hippocampi (Hp) and the frontal cortices (Cx) of offspring at PND7. The mRNA levels were measured using qRT-PCR with *n* = 6–8 in each group. The results are presented as the average fold change ± SEMs

Factor	Gene expression							
	Hp		Cx		Hp		Cx	
	kLPS	LPS	kLPS	LPS	kPoly	Poly	kPoly	Poly
***Cx3cl1***	1.01 ± 0.06	1.41 ± 0.17*	1.01 ± 0.07	1.09 ± 0.12	1.03 ± 0.12	0.92 ± 0.06	1.01 ± 0.06	0.90 ± 0.04
***Cx3cr1***	1.03 ± 0.09	1.31 ± 0.18	1.04 ± 0.10	0.98 ± 0.14	1.02 ± 0.09	1.06 ± 0.07	1.01 ± 0.06	0.88 ± 0.05
***Cd200***	1.00 ± 0.03	1.43 ± 0.19*	1.03 ± 0.11	1.21 ± 0.17	1.10 ± 0.23	0.89 ± 0.09	1.02 ± 0.10	0.73 ± 0.05*
***Cd200r***	1.07 ± 0.14	1.31 ± 0.18	1.09 ± 0.21	0.88 ± 0.26	1.09 ± 0.22	0.98 ± 0.15	1.06 ± 0.16	0.92 ± 0.06

*p < 0.05 vs. appropriate control (kLPS or kPoly)

### The impact of MIA generated by LPS and Poly I:C treatment on the protein levels of CX3CL1, CX3CR1, CD200 and CD200R in the hippocampi and the frontal cortices of offspring at PND7

**Fig. 2:**
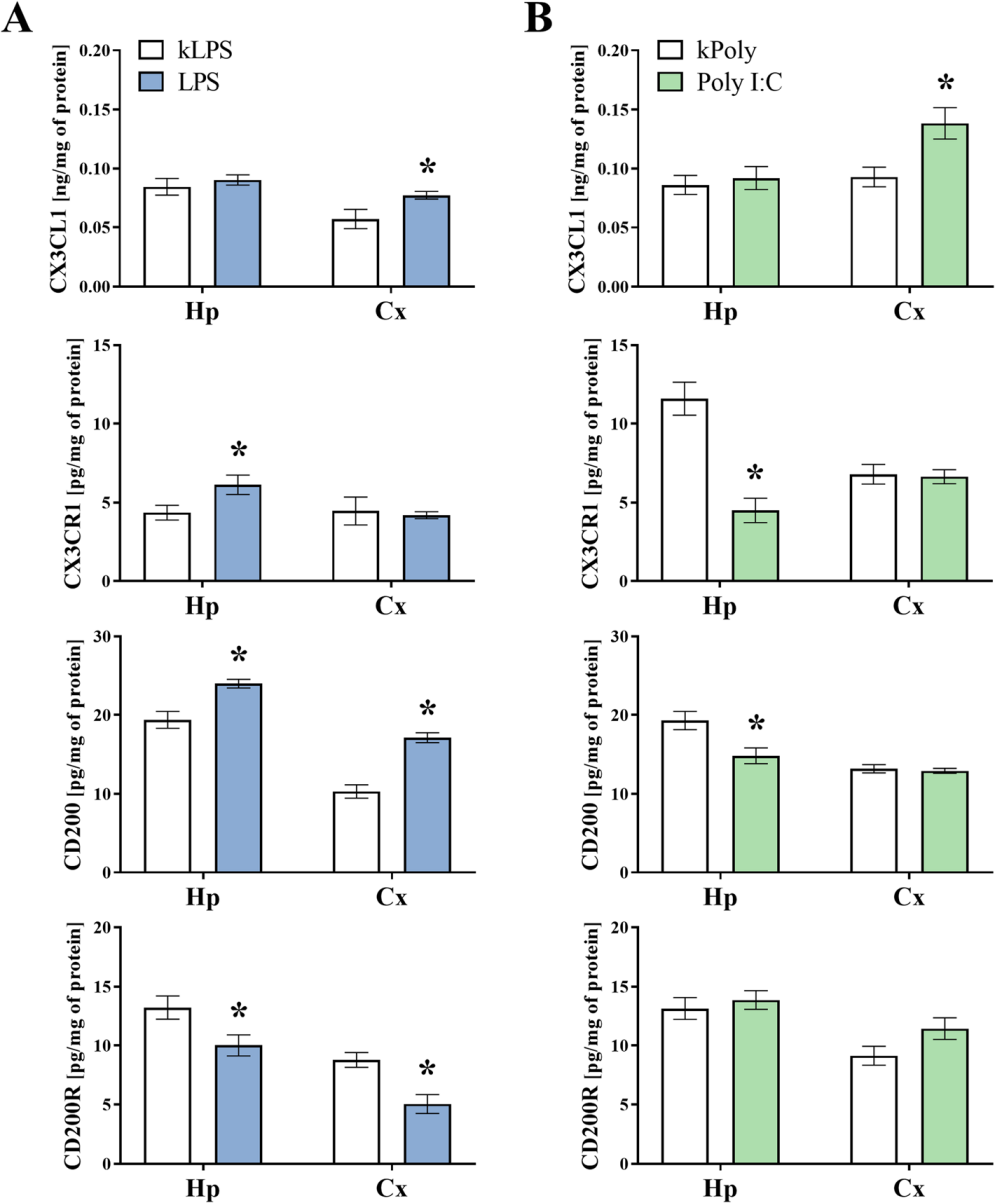
The effect of MIA induced by LPS (**a**) and Poly I:C (**b**) treatment on the protein levels of CX3CL1, CX3CR1, CD200 and CD200R in the hippocampi (Hp) and the frontal cortices (Cx) of PND7 offspring. *n* = 6–8 in each group. The results are presented as the means ± SEMs. *p < 0.05 vs. appropriate control (kLPS or kPoly)

Since the qRT-PCR analysis demonstrated that there were alterations caused by MIA induced by LPS and Poly I:C treatment in the neuron-microglia controlling systems in offspring at PND7, we next investigated the protein levels of CX3CL1, CX3CR1, CD200 and CD200R in the hippocampi and the frontal cortices of these animals (Fig. 2). A significant increase in CX3CR1 (*p* = 0.0413) and CD200 (*p* = 0.0030) was detected in the hippocampus of the LPS rats at PND7, while the CD200R level (*p* = 0.0307) was reduced in this structure. At the same time, we observed higher levels of both ligands: CX3CL1 (*p* = 0.0464) and CD200 (*p* < 0.0001), as well as decreased CD200R (*p* = 0.0025) in the frontal cortex of the prenatally LPS-exposed animals compared to what was observed in the kLPS group (Fig. 2a). The MIA with Poly I:C diminished CX3CR1 (*p* = 0.0003) and CD200 (*p* = 0.0129) levels in the hippocampus. Along with these results, we observed an increase in CX3CL1 (*p* = 0.0132) in the hippocampus in the Poly I:C offspring (Fig. 2b).

### Immunohistofluorescent staining of CX3CL1-CX3CR1 and CD200-CD200R localization on neurons and microglial cells in the hippocampi and the frontal cortices of offspring at PND7 after MIA generated by LPS and Poly I:C treatment

**Fig. 3:**
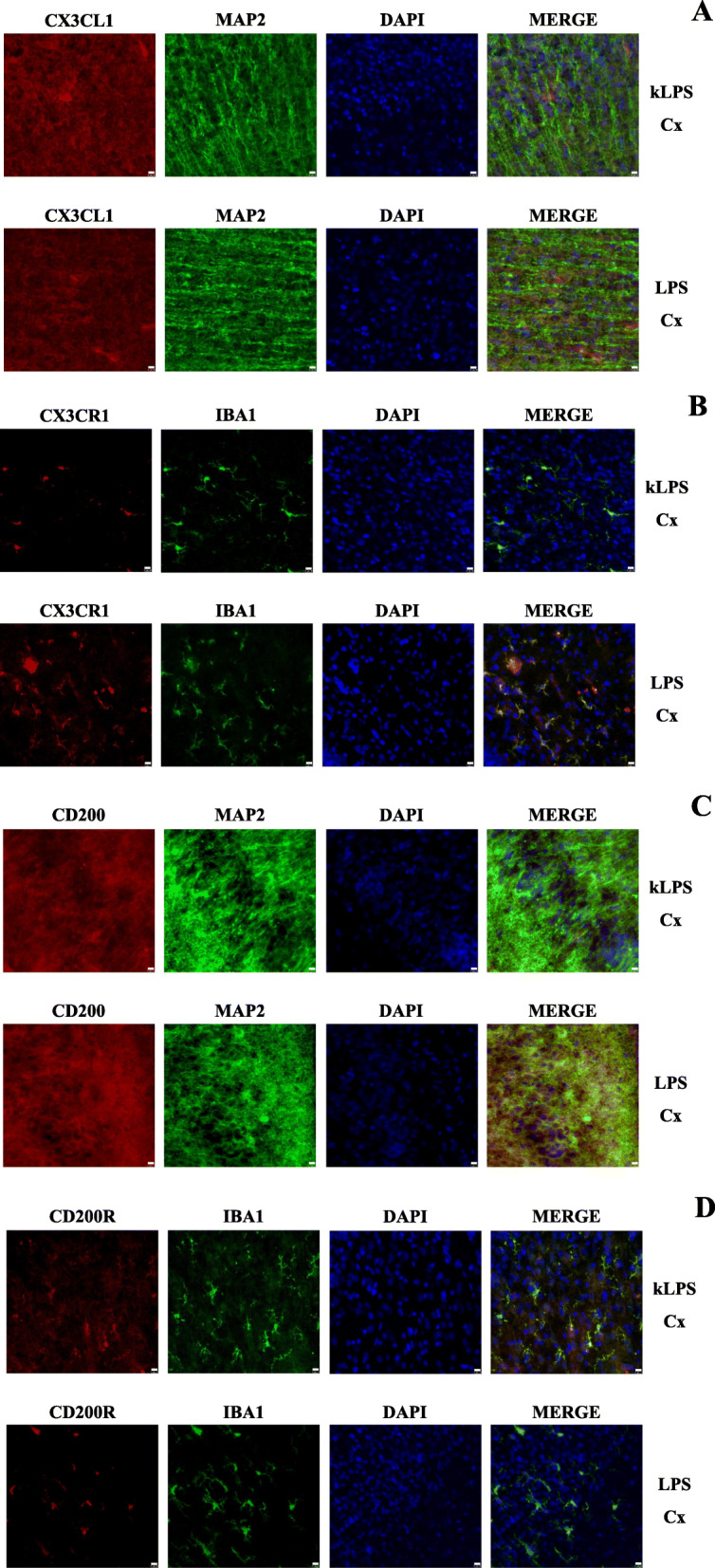
Immunohistofluorescent staining of CX3CL1-CX3CR1 (**a**, **b**) and CD200-CD200R (**c**, **d**) localization on neurons and microglial cells in the frontal cortex (Cx) of PND7 offspring after MIA induced by LPS treatment. Representative confocal images showing colocalization of CX3CL1/CD200 (red) immunoreactivity with MAP2 (green)-positive neurons and CX3CR1/CD200R (red) immunoreactivity with IBA1 (green)-positive microglial cells. *n* = 2 in each group. Magnification × 40 for all images. Scale bar (10 μm) is located in the bottom right corner of each image

**Fig. 4:**
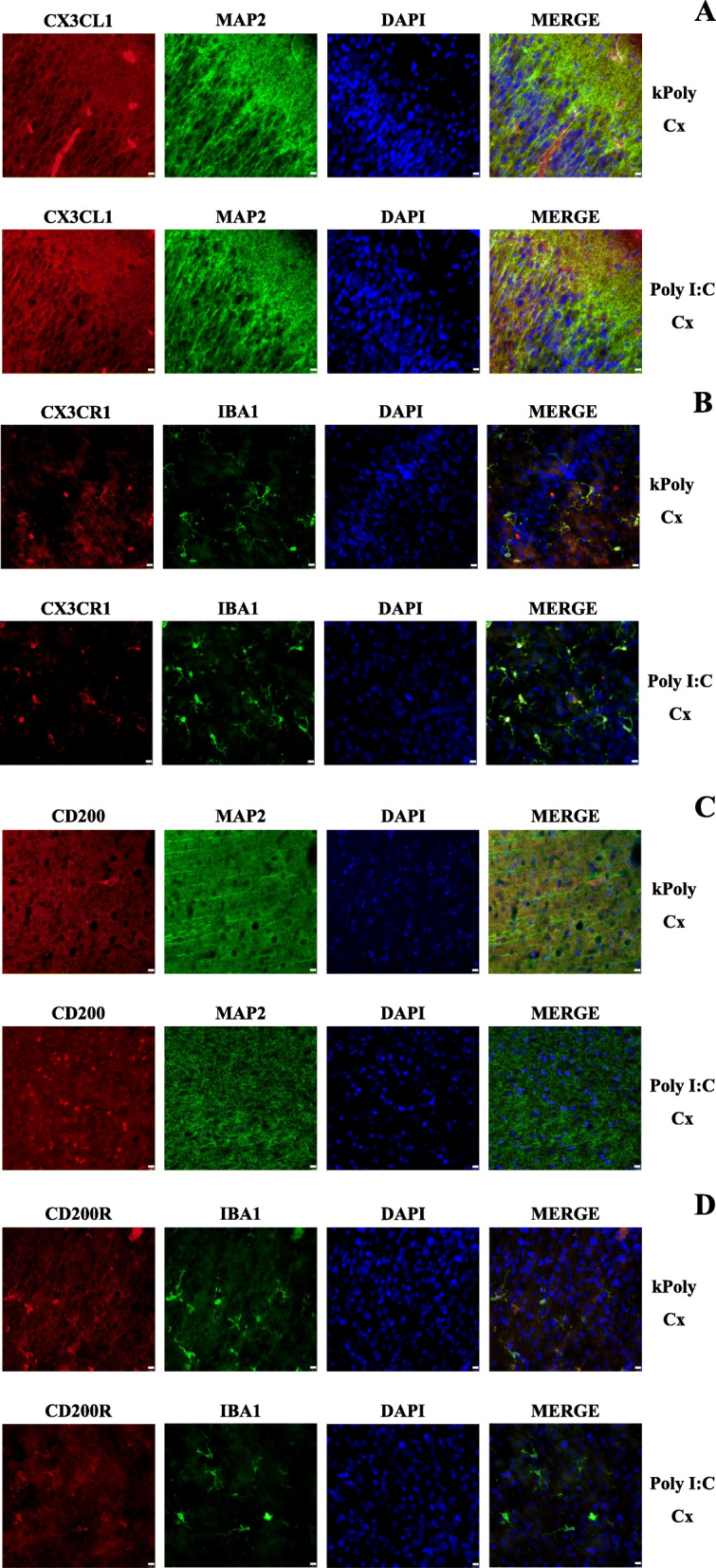
Immunohistofluorescent staining of CX3CL1-CX3CR1 (**a**, **b**) and CD200-CD200R (**c**, **d**) localization on neurons and microglial cells in the frontal cortex (Cx) of PND7 offspring after MIA induced by Poly I:C treatment. Representative confocal images showing colocalization of CX3CL1/CD200 (red) immunoreactivity with MAP2 (green)-positive neurons and CX3CR1/CD200R (red) immunoreactivity with IBA1 (green)-positive microglial cells. *n* = 2 in each group. Magnification × 40 for all images. Scale bar (10 μm) is located in the bottom right corner of each image

CX3CL1 and CD200 expression in the CNS has been mostly reported in neurons, while their receptors, CX3CR1 and CD200R, respectively, have been shown in microglia [44]. To confirm this phenomenon and to visualize whether treatment with LPS or Poly I:C during pregnancy affected these specific colocalizations in the brains of male rats at PND7, we performed immunohistofluorescent staining. In the DG, CA1 and CA3 fields of the hippocampus and the frontal cortex of the animals from the LPS and Poly I:C groups, we showed that exposure to neither immunostimulant influenced the colocalization of ligands or receptors with their specific cell types. For all studied regions of the male rat brains at PND7, we observed CX3CL1- and CD200-immunoreactive neurons and the expression of CX3CR1 and CD200R in microglia. Representative images of the staining are presented for the frontal cortex of LPS- (Fig. 3) and Poly I:C-treated animals (Fig. 4). Analogical images for the DG, CA1 and CA3 fields of the hippocampus are provided in Supplementary Figs. 1, 2, 3, 4, 5 and 6.

### The impact of MIA generated by LPS and Poly I:C treatment on IBA1 levels in the hippocampi and the frontal cortices of offspring at PND7

**Fig. 5:**
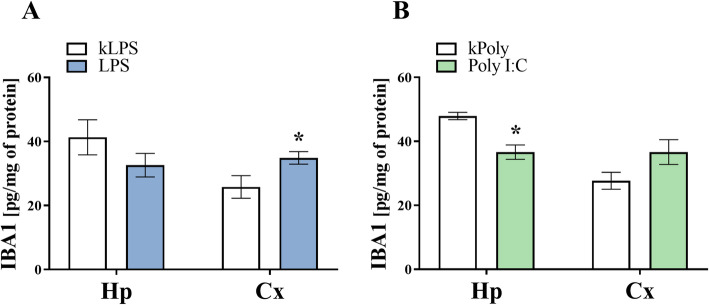
The effect of MIA induced by LPS (**a**) and Poly I:C (**b**) treatment on the protein level of IBA1 in the hippocampi (Hp) and the frontal cortices (Cx) of PND7 offspring. *n* = 6–7 in each group. The results are presented as the means ± SEMs. *p < 0.05 vs. appropriate control (kLPS or kPoly)

Since we confirmed the localization of CX3CR1 and CD200R in microglial cells, while biochemical analyses revealed changes in the levels of both receptors after MIA, next we assessed IBA1 levels in the hippocampi and the frontal cortices of the animals at PND7 (Fig. 5). The ELISA results showed an elevation in IBA1 (*p* = 0.0429) level in the frontal cortex of rats that were prenatally treated with LPS (Fig. 5a). In contrast, a diminished level of IBA1 (*p* = 0.0015) was found in the hippocampus of the offspring following Poly I:C treatment (Fig. 5b).

### The impact of MIA generated by LPS and Poly I:C treatment on the mRNA expression of microglial markers, pro- and anti-inflammatory factors in the hippocampi and the frontal cortices of offspring at PND7

CX3CL1-CX3CR1 and/or CD200-CD200R crosstalk plays a role in brain homeostasis due to the regulation of the “on-off” signal for microglial cell activation [45, 46]. Consequently, we explored the potential impact of these axes malfunctions on the microglia phenotype and immune response in the hippocampi and the frontal cortices of male offspring at PND7 after prenatal exposure to MIA with LPS and Poly I:C. As shown in Table 2, the levels of *Il-1β* (*p* = 0.0126) and *Tnf-α* (*p* = 0.0381) in the hippocampus but also *Cd40* (*p* = 0.0335) in the frontal cortex were significantly elevated in young offspring in the LPS group compared to those in the kLPS group. We did not observe changes in the other investigated markers of the pro-inflammatory phenotype of microglia (*MhcII*, *Cd68*, *Cd40*, *iNos*, *Il-6*) in this brain structure or in other M1-like factors in the frontal cortex of male offspring prenatally treated with LPS. Statistical analysis revealed that the anti-inflammatory phenotype of microglia in the hippocampus of the LPS group differed from that in the kLPS group (Table 2). There was a significant increase in the mRNA expression of *Arg1* (*p* = 0.0380), *Tgf-β* (*p* = 0.0354) and *Il-10* (*p* = 0.0466). The mRNA levels of *Igf-1* and *Il-4* in the hippocampus of male offspring at PND7 were not influenced by MIA induced with LPS. Additionally, we did not find any significant changes in the expression of any M2-like marker in the frontal cortex of the prenatally LPS-treated male animals. Among the tested markers of the M1-like microglial phenotype, qRT-PCR analysis showed elevated expression of *iNos* (*p* = 0.0498) in the hippocampus of the Poly I:C group (Table 2). The other examined M1-related factors (*MhcII*, *Cd68*, *Cd40*, *Il-1β*, *Tnf-α*, *Il-6*) were not affected by prenatal treatment with Poly I:C in this region of the brain in the male rats at PND7. In the frontal cortex of the Poly I:C group, MIA during pregnancy influenced the pro-inflammatory phenotype of microglia. We detected a reduction in *Cd40* (*p* = 0.0139) and a significant increase in *Il-6* (*p* = 0.0196) expression, whereas *MhcII*, *Cd68*, *iNos*, *Il-1β* and *Tnf-α* mRNA levels were not altered. The male offspring at PND7 in the Poly I:C group displayed no changes in M2-like factors in the hippocampus (Table 2). Regarding the results for the frontal cortex, analysis with Student’s *t* test demonstrated that *Tgf-β* (*p* = 0.0090) and *Il-10* (*p* = 0.0027) levels were significantly lower in the Poly I:C group than they were in the kPoly animals. Simultaneously, the mRNA expression of *Arg1* and *Igf-1* was not influenced in these groups.

**Table 2:** The effect of MIA induced by LPS and Poly I:C treatment on the gene expression of M1-like microglial markers and pro-inflammatory factors: *MhcII*, *Cd68*, *Cd40*, *iNos*, *Il-1β*, *Tnf-α* and *Il-6*, and M2-like microglial markers and anti-inflammatory factors: *Arg1*, *Igf-1*, *Tgf-β*, *Il-4* and *Il-10*, in the hippocampi (Hp) and the frontal cortices (Cx) of offspring at PND7. The mRNA levels were measured using qRT-PCR with *n* = 4–8 in each group. The results are presented as the average fold change ± SEMs

Factor	Gene expression							
	Hp		Cx		Hp		Cx	
	kLPS	LPS	kLPS	LPS	kPoly	Poly	kPoly	Poly
**M1-like phenotype**								
*MhcII*	1.03 ± 0.09	0.83 ± 0.06	0.98 ± 0.18	0.91 ± 0.11	1.02 ± 0.08	0.84 ± 0.12	1.17 ± 0.24	0.90 ± 0.11
*Cd68*	1.09 ± 0.07	1.44 ± 0.29	1.05 ± 0.13	1.02 ± 0.13	1.08 ± 0.18	0.76 ± 0.09	0.93 ± 0.03	0.81 ± 0.07
*Cd40*	1.01 ± 0.04	1.16 ± 0.14	0.97 ± 0.07	1.36 ± 0.14*	1.02 ± 0.09	0.92 ± 0.10	1.00 ± 0.04	0.80 ± 0.05*
*iNos*	0.96 ± 0.34	1.44 ± 0.21	0.92 ± 0.20	1.03 ± 0.21	0.81 ± 0.11	1.49 ± 0.26*	1.14 ± 0.21	1.21 ± 0.29
*Il-1β*	1.02 ± 0.08	1.79 ± 0.27*	1.02 ± 0.09	1.16 ± 0.12	1.04 ± 0.11	1.09 ± 0.07	1.03 ± 0.10	0.81 ± 0.05
*Tnf-α*	1.00 ± 0.03	1.63 ± 0.29*	1.02 ± 0.08	1.22 ± 0.14	1.08 ± 0.17	0.82 ± 0.06	1.04 ± 0.12	0.83 ± 0.07
*Il-6*	1.02 ± 0.08	1.24 ± 0.11	1.03 ± 0.10	1.20 ± 0.13	1.05 ± 0.11	0.92 ± 0.08	0.94 ± 0.12	1.37 ± 0.11*
**M2-like phenotype**								
*Arg1*	1.01 ± 0.05	1.59 ± 0.25*	1.02 ± 0.08	1.19 ± 0.12	1.05 ± 0.13	0.94 ± 0.08	1.04 ± 0.13	0.95 ± 0.09
*Igf-1*	1.01 ± 0.06	0.94 ± 0.02	1.00 ± 0.03	0.98 ± 0.06	1.03 ± 0.10	1.28 ± 0.09	1.02 ± 0.06	1.10 ± 0.05
*Tgf-β*	1.01 ± 0.05	1.64 ± 0.26*	1.08 ± 0.12	1.21 ± 0.09	1.03 ± 0.09	0.96 ± 0.06	1.02 ± 0.09	0.74 ± 0.04*
*Il-4*	1.05 ± 0.12	0.95 ± 0.11	1.21 ± 0.29	1.10 ± 0.25	1.03 ± 0.09	0.91 ± 0.10	Not detected	Not detected
*Il-10*	1.09 ± 0.18	1.77 ± 0.26*	0.86 ± 0.27	1.11 ± 0.19	1.04 ± 0.12	1.57 ± 0.21	1.06 ± 0.15	0.40 ± 0.07*

*p < 0.05 vs. appropriate control (kLPS or kPoly)

### The impact of MIA generated by LPS and Poly I:C treatment on the cytokine levels in the hippocampi and the frontal cortices of offspring at PND7

**Fig. 6:**
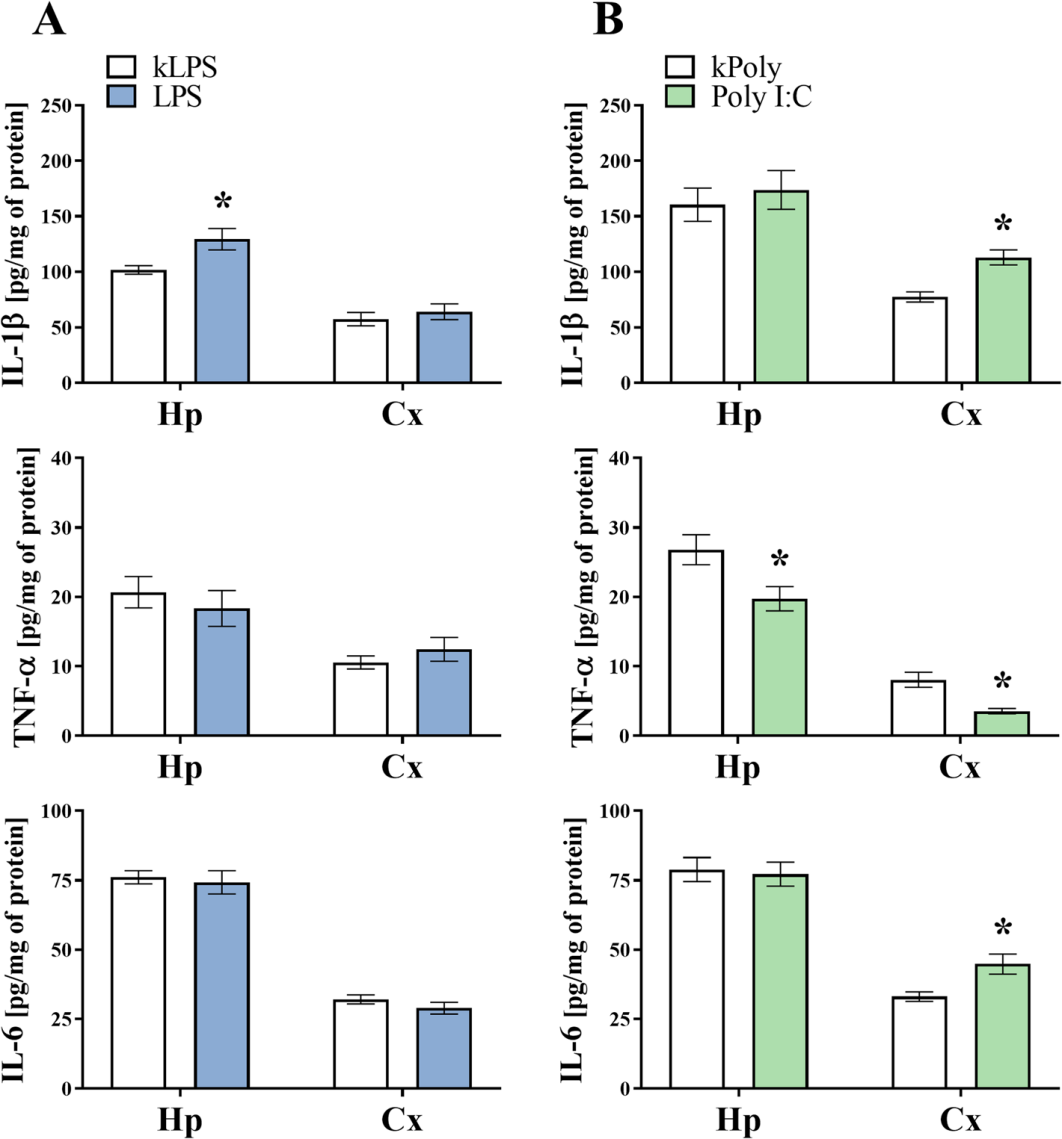
The effect of MIA induced by LPS (**a**) and Poly I:C (**b**) treatment on the protein levels of IL-1β, TNF-α and IL-6 in the hippocampi (Hp) and the frontal cortices (Cx) of PND7 offspring. *n* = 6–8 in each group. The results are presented as the means ± SEMs. *p < 0.05 vs. appropriate control (kLPS or kPoly)

**Fig. 7:**
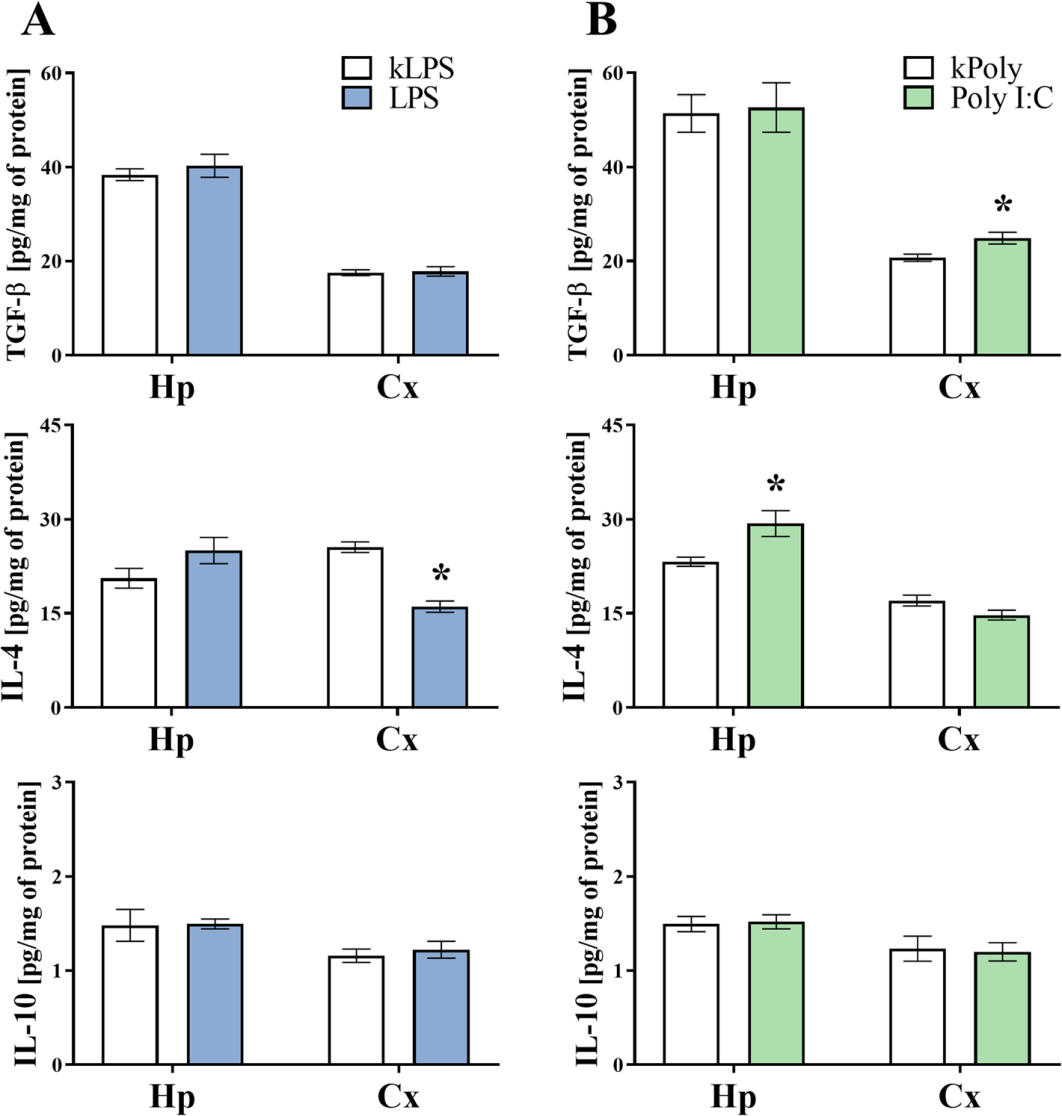
The effect of MIA induced by LPS (**a**) and Poly I:C (**b**) treatment on the protein levels of TGF-β, IL-4 and IL-10 in the hippocampi (Hp) and the frontal cortices (Cx) of PND7 offspring. *n* = 6–8 in each group. The results are presented as the means ± SEMs. *p < 0.05 vs. appropriate control (kLPS or kPoly)

The analysis of marker expression seems to be insufficient for capturing all changes in microglial reactivity, especially in MIA models, where subtle changes in microglial activity have been previously reported [47]. Accordingly, we also assessed the influence of MIA on the levels of pro- (IL-1β, TNF-α, IL-6) and anti-inflammatory (TGF-β, IL-4, IL-10) cytokines in both areas of the brain in male offspring at PND7 (Figs. 6 and 7). In the hippocampus of the LPS offspring, the levels of anti-inflammatory factors were not affected, while at the same time, IL-1β (*p* = 0.0168) level was raised (Figs. 6a and 7a). The ELISA results showed a decrease in IL-4 (*p* < 0.0001) level in the frontal cortex of the rats in the LPS group (Fig. 7a). Among the tested factors, an increase in IL-4 (*p* = 0.0251) and a reduction of TNF-α (*p* = 0.0275) were observed in the hippocampus of the Poly I:C offspring. Analysis with Student’s *t* test revealed that the protein levels of TGF-β (*p* = 0.0127), but also IL-1β (*p* = 0.0007) and IL-6 (*p* = 0.0109) were enhanced when TNF-α (*p* = 0.0019) declined in the frontal cortex of male animals at PND7 following prenatal exposure to Poly I:C compared to what we observed in the kPoly group (Figs. 6b and 7b).

### The impact of MIA generated by LPS and Poly I:C treatment on the behavioural parameters of adult male offspring

#### Prepulse inhibition of the acoustic startle response

**Fig. 8:**
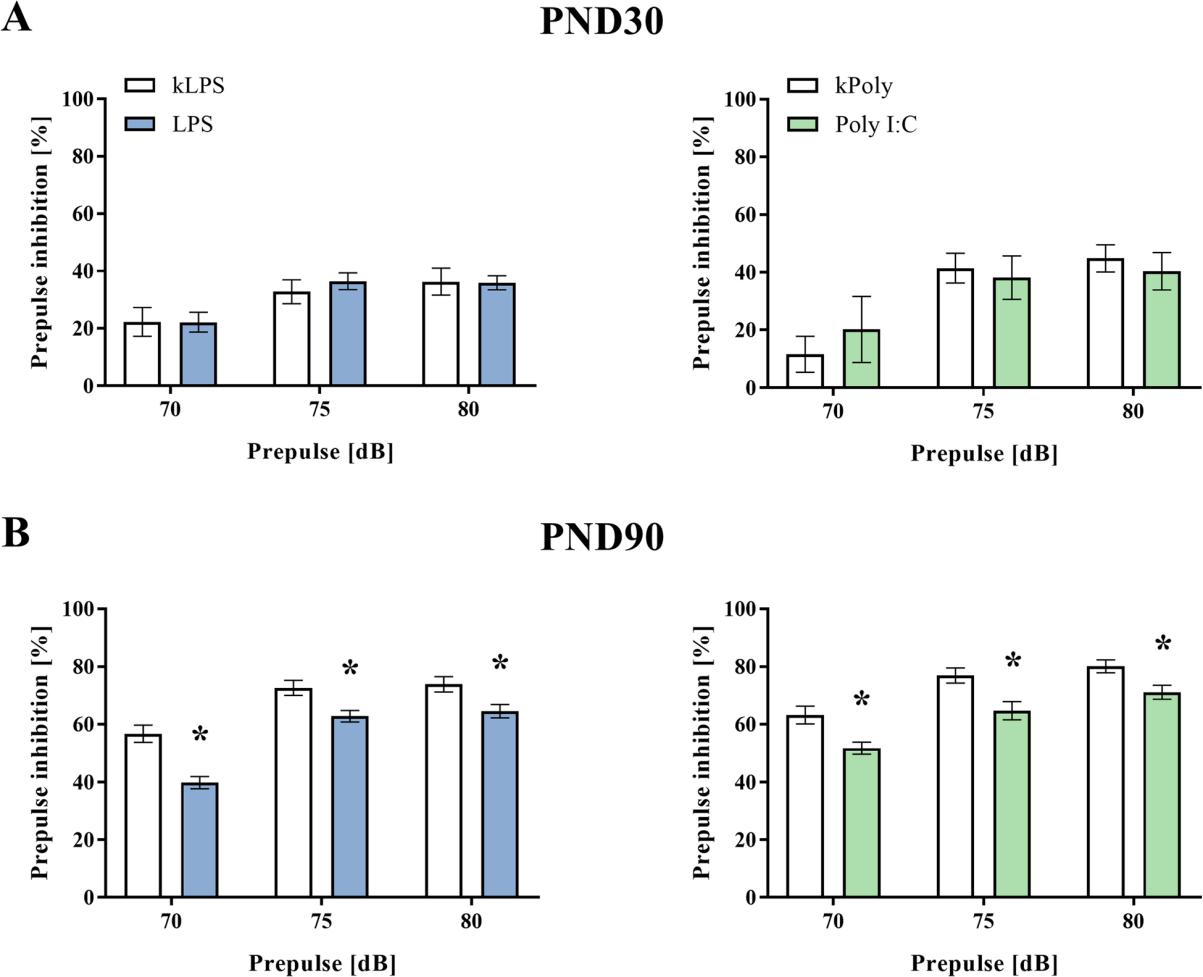
The effect of MIA induced by LPS and Poly I:C treatment on prepulse inhibition of the acoustic startle response (PPI) in male offspring at PND30 (**a**) and PND90 (**b**). *n* = 23 in the kLPS and LPS groups, *n* = 21 in the kPoly group and *n* = 14 in the Poly I:C group. The results are presented as the means of the percentage of PPI (%PPI) induced by each prepulse intensity ± SEMs. Data were calculated based on the average startle amplitudes (AVGs). *p < 0.05 vs. appropriate control (kLPS or kPoly)

Disturbed sensorimotor gating is one of the core behavioural features observed in schizophrenia, both for patients [48–50] and animal models of the disease [33, 51]. In Fig. 8, we show the impact of MIA induced by LPS and Poly I:C on the PPI response of adolescent (PND30) and adult (PND90) male rat offspring. The prenatal administration of LPS to pregnant dams did not disrupt PPI in adolescent male offspring (Fig. 8a), which corresponds with our previous data [32, 41]. Contrary, the adult animals in the LPS group displayed significant inhibition of sensorimotor gating compared to what we observed in the kLPS offspring for all tested prepulse intensities [70 (*p* < 0.0001), 75 (*p* = 0.0047) and 80 (*p* = 0.0115) dB] (Fig. 8b). Out of 39 adult rats in the LPS group, 23 (58%) showed a robust deficit in PPI. The same age-dependent effect was observed for the animals from the Poly I:C group, thus offspring at PND30 did not demonstrate any changes in PPI (Fig. 8a), whereas adult male rats (PND90) were characterized by a decrease in PPI compared to the kPoly offspring for all the prepulse groups [70 (*p* = 0.0092), 75 (*p* = 0.0054) and 80 (*p* = 0.0131) dB] (Fig. 8b). Out of 21 adult Poly I:C animals, 14 (67%) displayed an impairment of sensorimotor gating. These results confirm that MIA can lead to significant behavioural effects that shift over time.

#### Light-dark box test

**Fig. 9:**
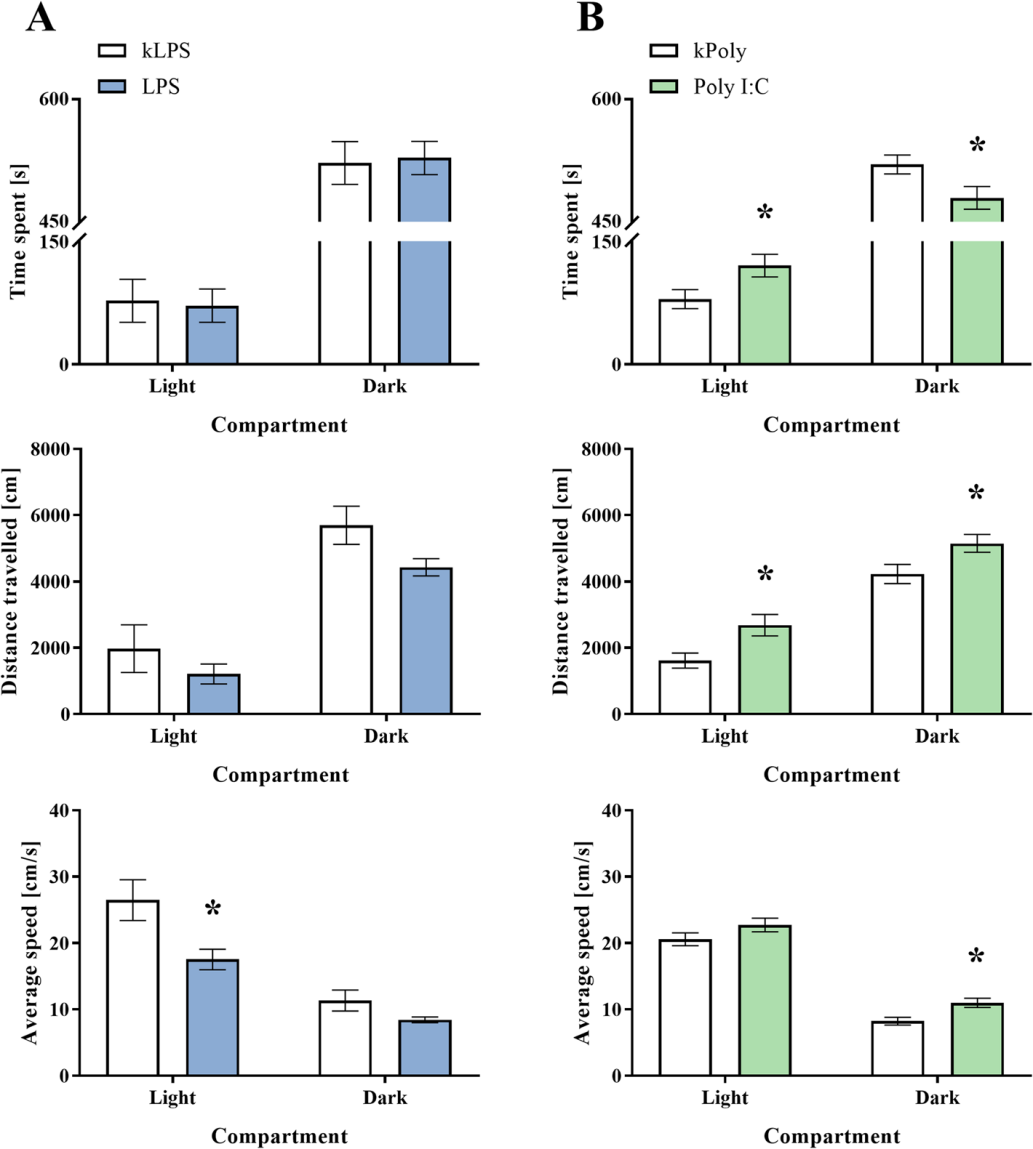
The effect of MIA induced by LPS (**a**) and Poly I:C (**b**) treatment on anxiety-like behaviour measured in the light-dark box test. *n* = 8–9 in the kLPS and LPS groups, *n* = 19–21 in the kPoly and Poly I:C groups. The results are presented as the means ± SEMs. *p < 0.05 vs. appropriate control (kLPS or kPoly)

Anxiety is one of the symptoms that sorely influences the quality of life in patients with schizophrenia [52]. We performed light-dark box test to assess whether the MIA induced by LPS and Poly I:C caused anxiety-like behaviour in adult rat offspring (PND88) (Fig. 9). Statistical analysis showed that male offspring from the LPS group did not differ from the kLPS animals in terms of time spent and distance travelled in the light part of the apparatus, but they did display a decrease in average speed (*p* = 0.0164). For the dark compartment, we observed only a decreasing tendency in distance travelled and average speed for the LPS rats, as well as no changes in time spent in this part of the experimental cage (Fig. 9a). The results of the light-dark box test carried out for the male Poly I:C offspring revealed that these rats spent more time (*p* = 0.0302) and covered a greater distance (*p* = 0.0120) than the kPoly group in the light compartment. The average speed of these animals was not changed by the prenatal treatment with Poly I:C. All three parameters measured in the dark compartment were significantly influenced by MIA for the Poly I:C group: time spent (*p* = 0.0120), distance travelled (*p* = 0.0247) and average speed (*p* = 0.0045) (Fig. 9b).

#### Social interaction test

**Fig. 10:**
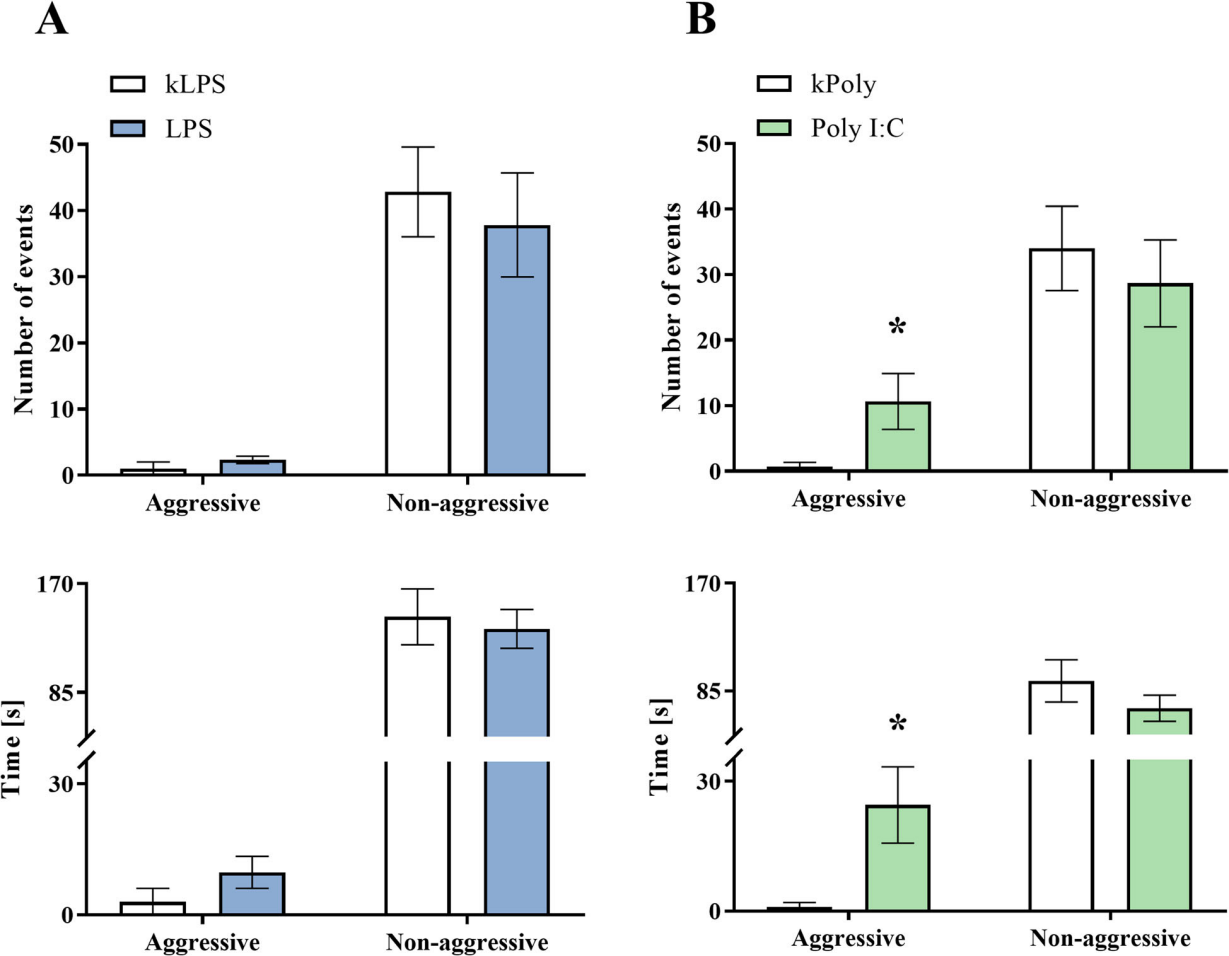
The effect of MIA induced by LPS (**a**) and Poly I:C (**b**) treatment on social (aggressive and non-aggressive) behaviour measured in the social interaction test. *n* = 6 in each group. The results are presented as the means ± SEMs. *p < 0.05 vs. appropriate control (kLPS or kPoly)

Social withdrawal, or asociality, is one of the primary negative symptoms of schizophrenia that has a significant impact on the functioning of the patients [53]. Unexpectedly, we showed that the treatment of pregnant rat females with LPS did not induce alterations either in the time or the number of non-aggressive and aggressive behaviours for male offspring at PND92 (Fig. 10a). In contrast to this finding, the social interactions of the animals in the Poly I:C group were significantly impaired. The rat offspring that were prenatally treated with Poly I:C were more aggressive, as evidenced by an increase in the time (*p* = 0.0248) and the number (*p* = 0.0433) of aggressive activities compared to the same measures in kPoly offspring (Fig. 10b).

### The impact of MIA generated by LPS and Poly I:C treatment on the protein levels of CX3CL1, CX3CR1, CD200 and CD200R in the hippocampi and the frontal cortices of adult offspring

**Fig. 11:**
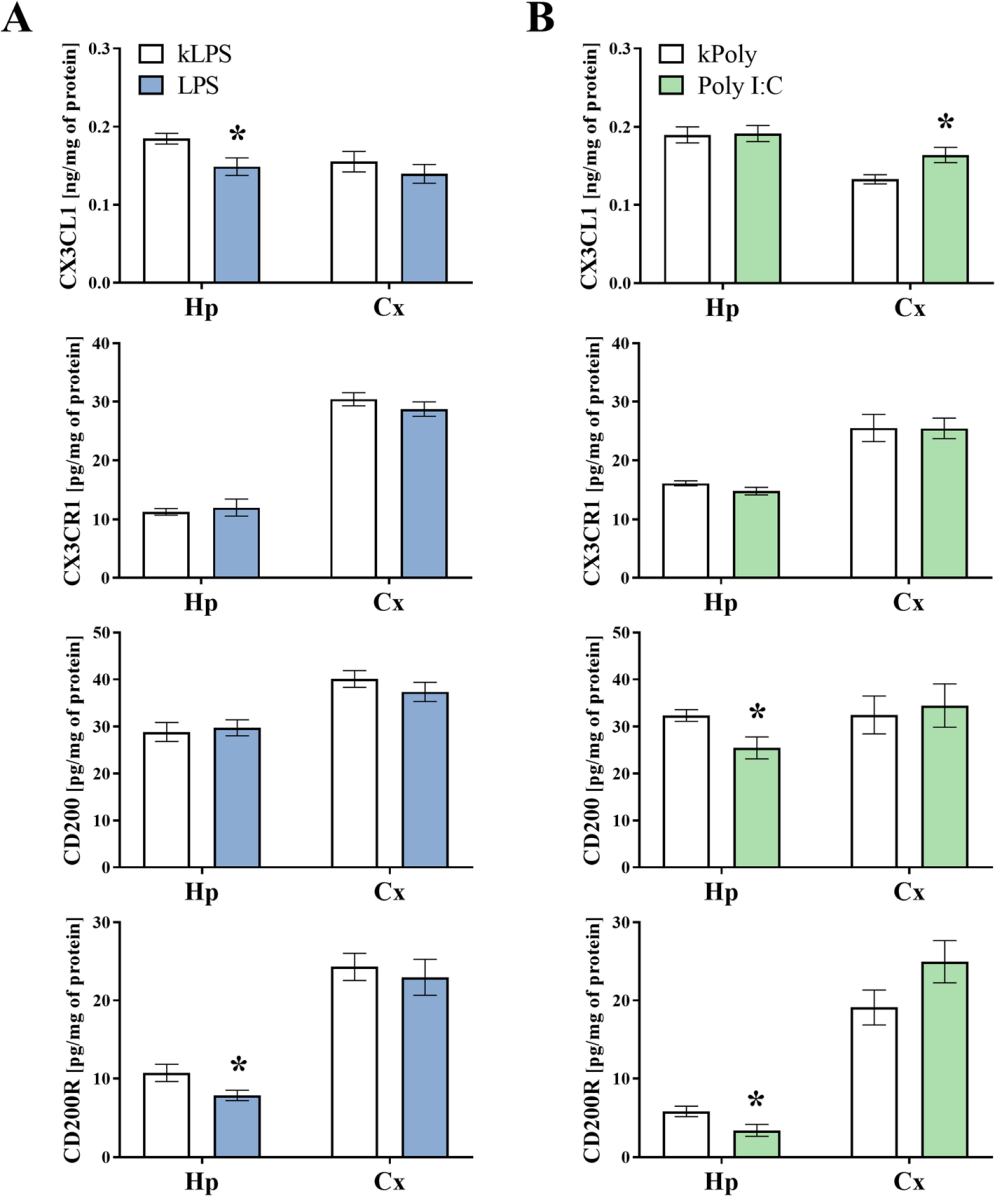
The effect of MIA induced by LPS (**a**) and Poly I:C (**b**) treatment on the protein levels of CX3CL1, CX3CR1, CD200 and CD200R in the hippocampi (Hp) and the frontal cortices (Cx) of PND93 offspring. *n* = 6–8 in each group. The results are presented as the means ± SEMs. *p < 0.05 vs. appropriate control (kLPS or kPoly)

In the next set of biochemical experiments, we investigated the protein levels of the systems controlling neuron-microglia interactions in the adulthood (PND93) (Fig. 11), since that was when the behavioural disturbances were present. The ELISA results showed a significant decrease in the protein levels of CX3CL1 (*p* = 0.0167) and CD200R (*p* = 0.0350) in the hippocampus of the LPS adult male rats when compared to that of the kLPS group. We did not observe any alterations in the frontal cortex of the LPS offspring (Fig. 11a). Analysis of the homogenates from the hippocampi of the Poly I:C group revealed that MIA diminished the levels of CD200 (*p* = 0.0165) and CD200R (*p* = 0.0361). Contrary, in the frontal cortex of adult male rats after prenatal treatment with Poly I:C, only CX3CL1 (*p* = 0.0135) level was elevated comparing to the kPoly group (Fig. 11b).

### The impact of MIA generated by LPS and Poly I:C treatment on IBA1 levels in the hippocampi and the frontal cortices of adult offspring

**Fig. 12:**
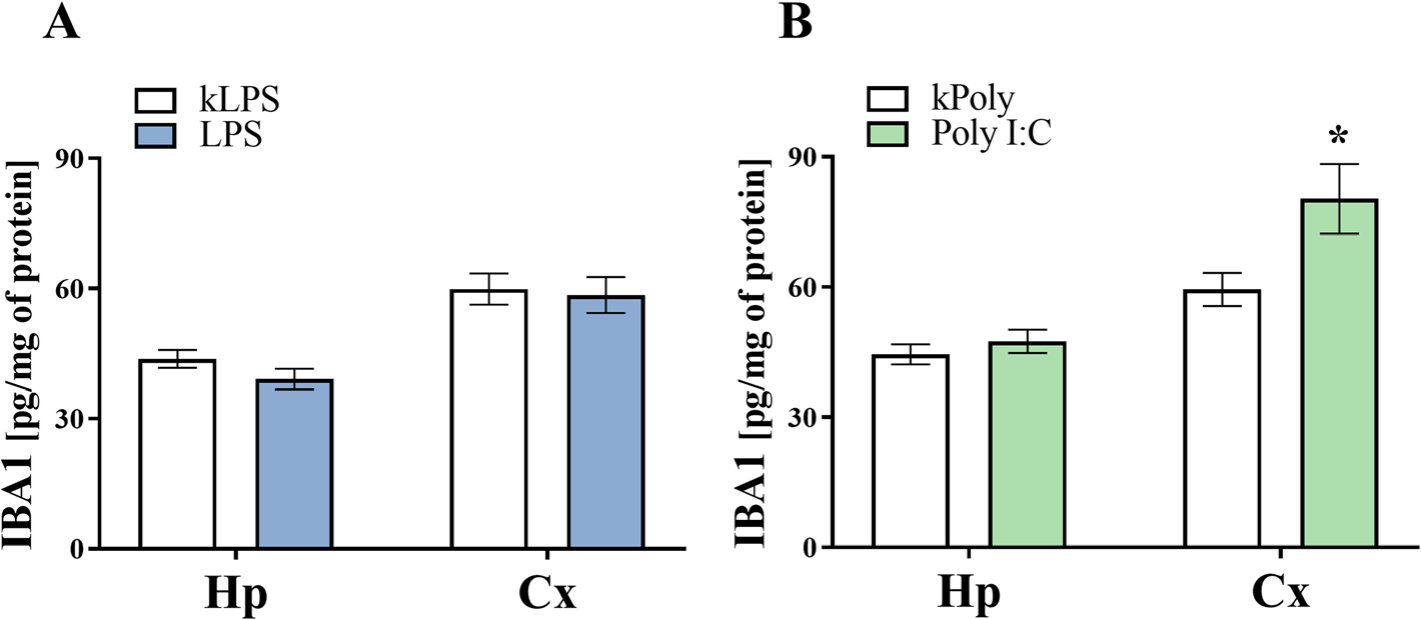
The effect of MIA induced by LPS (**a**) and Poly I:C (**b**) treatment on the protein level of IBA1 in the hippocampi (Hp) and the frontal cortices (Cx) of PND93 offspring. *n* = 6–9 in each group. The results are presented as the means ± SEMs. *p < 0.05 vs. appropriate control (kLPS or kPoly)

Having found that the homeostasis of neuron-microglia communication was somewhat impaired in the brains of offspring at PND93, we wanted to determine whether the observed changes were related to IBA1 levels. We did not find any changes in IBA1 levels in the hippocampi or frontal cortices of the animals from the LPS group at PND93 (Fig. 12a). Regarding the Poly I:C offspring, the ELISA analysis revealed an elevation in IBA1 (*p* = 0.0257) level in the frontal cortex of these animals (Fig. 12b).

## Discussion

There is a growing body of evidence that in the CNS, preservation of proper neuron-microglia interactions is crucial for brain development and homeostasis [1, 2, 54]. This dynamic crosstalk is under the control of endogenous factors, including the CX3CL1-CX3CR1 and CD200-CD200R axes. However, despite the pivotal role of these signalling pathways in brain homeostasis, very little is known about the effect of MIA on both systems in the context of schizophrenia pathogenesis.

Therefore, the most important finding presented in our study is that MIA with both LPS and Poly I:C affected CX3CL1-CX3CR1 and/or CD200-CD200R signalling in early life, which is the period when crucial neurodevelopmental processes occur. Moreover, we provided evidence that MIA disturbed the expression of microglial markers and the profile of cytokines released in the brains of young offspring. The observed effect was long-termed as it was present also in adulthood, in the period when behavioural schizophrenia-like disturbances arose.

### Maternal immune activation with LPS and Poly I:C leads to neuron-microglia communication changes in young male rat offspring

Here, we report that in the frontal cortex of young offspring, the protein level of CX3CL1 was elevated after prenatal exposure to both LPS and Poly I:C. In the hippocampus, MIA evoked by LPS administration induced the upregulation of *Cx3cl1* expression. At the same time, in the hippocampus, we noted changes in the level of CX3CR1 (namely, an increase after MIA induced by LPS and a decrease after Poly I:C treatment), which indicates that the immunogen type determines the changes in this receptor level. In early brain development, efficient CX3CL1-CX3CR1 crosstalk participates in the regulation of neuronal cell number by phagocytosis (e.g., hippocampal neurons) or via impact on the emergence of connectivity by promoting outgrowth of axonal tracts (e.g., dopaminergic) [55]. Although CX3CL1 is constitutively expressed in neurons [19, 56], some data demonstrated that astrocytes could also be a source of this ligand [11, 57]. However, the excessive increase in CX3CL1 in MIA models observed in our study should not be explained by this phenomenon because immunohistofluorescent staining demonstrated that after MIA, CX3CL1 in the frontal cortex is expressed by neurons (Figs. 3 and 4). Interestingly, in endothelial cells, excessive release of CX3CL1 may be induced by pro-inflammatory cytokines, particularly IL-1β and TNF-α [58]. Therefore, the increase in IL-1β level after prenatal exposure to Poly I:C may, to some extent, result in excessive secretion of CX3CL1 at PND7. Moreover, malfunction in the CX3CL1 shedding from the membrane, induced by MIA, should also be considered. ADAM 10 metalloprotease, which cleaves CX3CL1 into a secreted form, is the main involved in CX3CL1 release under pro-inflammatory conditions in the brain [59, 60]. This dynamic proteolytic cleavage of CX3CL1 from neuronal membranes and subsequent chemoattraction of reactive immune cells may represent an early event in the inflammatory response to neuronal injury [61]. The CX3CL1-CX3CR1 axis is also crucial for controlling the microglial phenotype and its proper functioning during early development. For example, the highest level of CX3CL1 in the brain is observed during embryonic and postnatal maturation periods, and the expression declines with age [62]. It has been found that CX3CL1 regulates the distinctive colonization pattern of microglia populations [63], while dysfunction in CX3CL1 signalling impairs this process in the cortex and in the hippocampus [64].

In our study, a modulating effect of MIA on CX3CR1 level was found only in the hippocampus of young offspring. In this structure, the elimination of synapses is highly dependent on CX3CR1, since mice lacking this receptor displayed increased hippocampal dendritic spine density [65]. This phenomenon was accompanied by synaptic characteristics reminiscent of immature connectivity and weak synaptic transmission [65]. In line with the above results, CX3CR1 expression on microglial cells, visualized by us using CX3CR1/IBA1 double staining, was decreased in the hippocampus of prenatally Poly I:C-treated offspring. Therefore, changes generated by MIA can be responsible for similar deficits in synapses, although such conclusions require further studies, especially in the context of the observed reduction of IBA1. Since the microglial population is believed to be composed of long-lived cells, some disturbances evoked by MIA in CX3CL1-CX3CR1 signalling shown in our study may alter the microglial trajectory and neuronal function in adulthood [66].

To further characterize the involvement of MIA in neuron-microglia communication in young offspring, we evaluated the impact of prenatal stimulation with LPS and Poly I:C on CD200-CD200R signalling. We clearly demonstrated that MIA induced by LPS caused significant alterations in the CD200-CD200R interaction both in the hippocampus and in the frontal cortex of young offspring, mostly at the protein level. In fact, the expression of CD200 was boosted, whilst the CD200R, which is located on microglia (Figs. 3 and 4), level declined in both brain structures. During homeostasis, CD200-CD200R signalling has a recognized effect on the microglia population because it modulates the proliferation and apoptosis of the cells [67, 68]. CD200-CD200R axis controls microglial migration [69] and phagocytosis [70], while dysfunction of this pathway leads to microglial disinhibition. *Cd200*-deficient animals showed an increased production of iNOS [67], higher expression of TNF-α as well as IFN-γ in the hippocampus [24], which in turn had a negative effect on neuronal function [71]. The lack of CD200R produced an exaggerated response of microglia, i.e., microglia priming, which is linked to proliferation, altered morphology and production of pro-inflammatory factors [72].

Considering the above data highlighting that the CD200-CD200R axis is an inhibitory system [23], we next explored the impact of prenatal LPS administration on the microglia phenotypes and cytokine levels in the hippocampus and the frontal cortex of young offspring. Thus, after prenatal LPS treatment, our studies revealed a dual increase in gene expression in the hippocampus, including genes related not only to anti- (*Arg-1*, *Tgf-β*, *Il-10*) but also to the pro-inflammatory phenotype (*Il-1β*, *Tnf-α*) with a concomitant increase in the protein level of IL-1β. On the other hand, in the frontal cortex of young offspring, the CD200-CD200R axis deficits were accompanied by *Cd40* upregulation and significantly diminished IL-4 release. Although there are still no data on the effect of MIA on these parameters at PND7, it has already been shown that anti-inflammatory cytokines play a crucial role in CD200-CD200R axis regulation. Among other factors, CD200R expression is strongly upregulated by IL-4 and IL-13 [73], which share common receptor [74, 75], while in *Il-4*-/- knockout mice, the level of CD200R in the brain is decreased [76]. Overall, these data suggest that MIA produced by LPS, through CD200-CD200R axis dysfunction, could change the immune status of the brain in young offspring.

Continuing our study, we showed that the consequence of MIA evoked by prenatal Poly I:C treatment on CD200-CD200R crosstalk was less pronounced and expressed as ligand changes, specifically the downregulation of *Cd200* in the frontal cortex and CD200 in the hippocampus of young animals. The unfavourable influence of Poly I:C on the CD200-CD200R system during pregnancy has been reported by Lin et al. [77]. Based on these data, the deficit of CD200 in Poly I:C-treated offspring can have harmful consequences on neuronal function because, as demonstrated, CD200 binding to CD200R produces an indirect protective effect on neurons and other cells in the brain [78]. As the expression of CD200 also affects CD200R activation, indirect effects derived from deficits in CD200 levels may influence the microglia phenotype [79]. Therefore, we next explored the impact of Poly I:C on the expression of various genes related to classical and alternative microglial activation and cytokines release in both brain areas of young offspring. Although IBA1 level was reduced in the hippocampus, we showed a rise in *iNos* expression. Recently, Liu et al. [80] demonstrated the functional role of *N*-glycosylation of CD200R in classical microglia activation. A mutation at asparagine 44 (N44) disrupted CD200-CD200R crosstalk and facilitated classical microglia activation characterized by the expression of M1-like phenotype markers, including *iNos*. On the other hand, considering the decreased TNF-α, while IL-4 raised protein levels in the hippocampus at PND7, only the modulatory properties of prenatal Poly I:C treatment on the immune response in the neonatal brain can be suggested. Assessing the changes in the frontal cortex, we observed that exposure to Poly I:C leads to upregulation of IL-1β and IL-6 and downregulation of anti-inflammatory gene expression (*Tgf-β* and *Il-10*). It may be suggested that the changes caused by MIA in CX3CL1-CX3CR1 and/or CD200-CD200R interactions and in the developmental trajectory of microglia from early stages to adulthood depend, at least in part, on the production of cytokines that act later in time. Indeed, specific cytokines have been found to play a critical role both in Poly I:C- and LPS-based MIA. In our study, a robust increase in IL-6 levels was observed only in offspring after prenatal Poly I:C treatment. In line with the abovementioned fundings, systemic IL-6 injection during pregnancy was able to generate similar behavioural deficits as Poly I:C, while the injection with an IL-6-blocking antibody could prevent MIA-induced behavioural changes [81]. Complementary deletion of the IL-6 receptor from the placenta could prevent MIA-induced behavioural deficits in offspring [82], while the presence of IL-6 in maternal blood was sufficient to induce social impairment in the offspring [83]. When IL-1β and IL-6 levels were simultaneously increased, a reduction of the elevated IL-1β, which was also observed in our study in the frontal cortex of offspring prenatally exposed to Poly I:C, did not alleviate deficits in this model [83]. Since the administration of an IL-1R antagonist prevents changes in the placenta and protects prenatally LPS-exposed offspring against motor dysfunction, it can suggest that IL-1β upregulation, shown by us in offspring, is important for the induction of deficits in the MIA model based on LPS treatment [84].

### Maternal immune activation with LPS and Poly I:C leads to schizophrenia-like behaviour and neuron-microglia communication changes in adult male rat offspring

Disturbed sensorimotor gating is one of the behavioural features observed in patients with schizophrenia [48–50] and in animal models [33, 51, 85–87]. The PPI deficit has been proposed as an experimental model of informative overflow resulting from an inability to properly perceive and filter information as it appears [88]. Our previously published data demonstrated age-dependent alterations in the amplitude of the startle reflex and deficits in PPI evoked by LPS in rats [32, 40, 41]. We confirmed these observations and what is more, also those showing that MIA induced by Poly I:C led to deficits in PPI in adult offspring [89, 90]. However, these alterations were absent at PND30, which indicated that PPI changes caused by Poly I:C were also age-dependent. The functional basis of PPI is regulated by the brainstem, but it is highly modulated by cerebral (including frontocortical) inputs [91, 92] as well as dopamine [93, 94] and serotonin transmission [95, 96]. Since CX3CL1 signalling participates in the regulation of these neurotransmitters [97, 98], the question arises whether and how the increase in CX3CL1 level in the frontal cortex of offspring during the neurodevelopmental period could affect PPI deficits. Considering that the lack of CX3CL1 signalling and weakened crosstalk between neurons and microglia affects transmission efficiency in synapses in an adult brain [99], a significance of changes in the level of this ligand in adult animals can be considered. In prenatally LPS-treated offspring, we also demonstrated dysfunction of the CD200-CD200R interaction and IL-1β upregulation, which, in turn, both potentiate dopaminergic-induced neurodegeneration [100] and contribute to cognitive impairment [101]. In line with the above results, the most intriguing observation indicates that MIA evoked by LPS treatment dysregulated these parameters in both studied brain structures in young offspring, but CD200R deficit was also observed in the hippocampus of adult offspring in both MIA models. Hence, the changes in the CD200-CD200R axis seem to be long term and therefore should be included while analysing the mechanism of behavioural deficit development.

Anxiety and social withdrawal are symptoms commonly observed in patients with schizophrenia [52, 102]. Concerning these behavioural characteristics, the repeated administration of LPS during pregnancy resulted only in a tendency towards anxious behaviour in adult offspring, which was indicated by the reduced light-dark box exploration. Similar observations have been reported for the progeny of mouse dams injected with LPS at GD9 when challenged in adulthood [103]. Also, the offspring prenatally exposed to intrauterine inflammation [104] and vaginitis [105], which are both induced by LPS treatment, manifested anxiety-related behaviours. Additionally, in our study, the LPS-treated adult male rats did not display any changes in social interactions. This finding contrasts with the data described previously by our group [85], which could result from differences in protocols applied [43], with previously based on the assessment of social behaviour using the resident-intruder paradigm. At the same time, for adult male animals from the Poly I:C group, we revealed an anxiolytic phenotype, which may indicate that prenatal contact with Poly I:C resulted in an elevation of psychomotor activity and apparently reduced the animal’s innate fear of open spaces, which facilitated exploration. A similar tendency was observed by Vorhees et al. [106]. A potential explanation for these changes is difficult to provide; nonetheless, to some extent, the reduced level of CX3CR1 observed in the hippocampus of these young rats should be considered. The results of Bachstetter et al. [107] have suggested that CX3CL1-CX3CR1 signalling has a regulatory role in modulating hippocampal neurogenesis. In addition, hippocampal neurogenesis has been implicated, for example, in stress resiliency in relation to anxiety disorder [108]. Research on 4-month-old female *Cx3cr1*-/- mice has revealed that these knockout animals have a hyperactive, anxiolytic-like phenotype [109]. In our study, in the adult males exposed to Poly I:C, the social interactions were shifted towards enhanced aggressive behaviour. Similarly, experimental data from animal models wherein Poly I:C was administrated to pregnant mice have highlighted the presence of an aggressive phenotype in adult offspring [110, 111]. Therefore, the diminished hippocampal CX3CR1 level, which we observed in prenatally Poly I:C-treated young offspring, opens the possibility that disruptions of microglia-mediated activity could contribute to neurodevelopmental deficits manifested as behavioural schizophrenia-like changes in adulthood.

### Limitations of our study

One of the limitations in the present study is the fact that our research was concentrated only on male offspring of Wistar rats. The main reason came from the evidence that the incidence of schizophrenia is significantly higher in men than it is in women [112–114]. Additionally, in terms of disease onset, symptom severity, neuropathology and response to treatment, there are notable differences between men and women suffering from this psychiatric disease [115, 116]. The experimental data have indicated sex as a moderating factor in schizophrenia for a wide range of biochemical characteristics, including glutamatergic transmission [117], the GABA-ergic system [85] and, what is crucial in the context of this article, the microglial phenotype [118–121]. Among other mechanisms, increased microglial reactivity to prenatal immune challenges, determining disease outcome in adulthood, shows a robust sex bias [118]. Therefore, it cannot be excluded that the picture of MIA-induced effects on the CX3CL1-CX3CR1 and CD200-CD200R axes and microglial phenotype may be different in females and thus should be a focus of further studies.

As the second limitation may be considered the fact that we performed the immunohistofluorescent stainings only to visualize the localization of the ligands (CX3CL1, CD200) and the receptors (CX3CR1, CD200R) on different cell types in brain areas of the young offspring from the control groups and the animals prenatally exposed to MIA. In our study, the effect of MIA on the ligand-receptor signalling pathways was assessed by applying the biochemical techniques (qRT-PCR, ELISA) in the homogenates of whole structures (hippocampus, frontal cortex). Nevertheless, we are fully aware that the quantitive analysis of the images could give additional important information about the cell- and area-dependent impact of MIA and should be considered in future studies.

## Conclusions

Our data demonstrated that MIA with LPS and Poly I:C alters developmental trajectories in neuron-microglia communication, especially the CX3CL1-CX3CR1 and CD200-CD200R systems, in the brains of young offspring. In addition, our data suggest that MIA-induced abnormalities may represent an important mechanism for the emergence of functional microglial changes associated with imbalances in the offspring immune system.

Our data do not provide a direct link between the altered CX3CL1-CX3CR1 and/or CD200-CD200R axes in young offspring and the occurrence of behavioural disturbances in adulthood. However, our results highlight for the first time that neuron-microglia abnormalities emerging after prenatal immune challenge may affect early neurodevelopment of the brain of young male offspring. Whether neuron-microglia changes generated by MIA are a potential mechanism of brain pathology, leading to schizophrenia, requires further research.

## Supplementary information

**Additional file 1: Table S1.** A list of genes (with corresponding catalogue numbers of TaqMan probes) examined in the hippocampi and the frontal cortices of male offspring at PND7 using qRT-PCR. *B2m* or *Hprt* were used as the reference genes.

**Additional file 2: Figure S1.** Immunohistofluorescent staining of CX3CL1-CX3CR1 (A, B) and CD200-CD200R (C, D) localization on neurons and microglial cells in the DG of the hippocampus of PND7 offspring after MIA induced by LPS treatment. Representative confocal images showing colocalization of CX3CL1/CD200 (red) immunoreactivity with MAP2 (green)-positive neurons and CX3CR1/CD200R (red) immunoreactivity with IBA1 (green)-positive microglial cells. *n* = 2 in each group. Magnification: 40x for all images. Scale bar (10 μm) is located in the bottom right corner of each image.

**Additional file 3: Figure S2.** Immunohistofluorescent staining of CX3CL1-CX3CR1 (A, B) and CD200-CD200R (C, D) localization on neurons and microglial cells in the DG of the hippocampus of PND7 offspring after MIA induced by Poly I:C treatment. Representative confocal images showing colocalization of CX3CL1/CD200 (red) immunoreactivity with MAP2 (green)-positive neurons and CX3CR1/CD200R (red) immunoreactivity with IBA1 (green)-positive microglial cells. *n* = 2 in each group. Magnification: 40x for all images. Scale bar (10 μm) is located in the bottom right corner of each image.

**Additional file 4: Figure S3.** Immunohistofluorescent staining of CX3CL1-CX3CR1 (A, B) and CD200-CD200R (C, D) localization on neurons and microglial cells in the CA1 field of the hippocampus of PND7 offspring after MIA induced by LPS treatment. Representative confocal images showing colocalization of CX3CL1/CD200 (red) immunoreactivity with MAP2 (green)-positive neurons and CX3CR1/CD200R (red) immunoreactivity with IBA1 (green)-positive microglial cells. *n* = 2 in each group. Magnification: 40x for all images. Scale bar (10 μm) is located in the bottom right corner of each image.

**Additional file 5: Figure S4.** Immunohistofluorescent staining of CX3CL1-CX3CR1 (A, B) and CD200-CD200R (C, D) localization on neurons and microglial cells in the CA1 field of the hippocampus of PND7 offspring after MIA induced by Poly I:C treatment. Representative confocal images showing colocalization of CX3CL1/CD200 (red) immunoreactivity with MAP2 (green)-positive neurons and CX3CR1/CD200R (red) immunoreactivity with IBA1 (green)-positive microglial cells. *n* = 2 in each group. Magnification: 40x for all images. Scale bar (10 μm) is located in the bottom right corner of each image.

**Additional file 6: Figure S5.** Immunohistofluorescent staining of CX3CL1-CX3CR1 (A, B) and CD200-CD200R (C, D) localization on neurons and microglial cells in the CA3 field of the hippocampus of PND7 offspring after MIA induced by LPS treatment. Representative confocal images showing colocalization of CX3CL1/CD200 (red) immunoreactivity with MAP2 (green)-positive neurons and CX3CR1/CD200R (red) immunoreactivity with IBA1 (green)-positive microglial cells. *n* = 2 in each group. Magnification: 40x for all images. Scale bar (10 μm) is located in the bottom right corner of each image.

**Additional file 7: Figure S6.** Immunohistofluorescent staining of CX3CL1-CX3CR1 (A, B) and CD200-CD200R (C, D) localization on neurons and microglial cells in the CA3 field of the hippocampus of PND7 offspring after MIA induced by Poly I:C treatment. Representative confocal images showing colocalization of CX3CL1/CD200 (red) immunoreactivity with MAP2 (green)-positive neurons and CX3CR1/CD200R (red) immunoreactivity with IBA1 (green)-positive microglial cells. *n* = 2 in each group. Magnification: 40x for all images. Scale bar (10 μm) is located in the bottom right corner of each image.

## Data Availability

All data supporting the conclusions of this manuscript are provided in the text, figures, tables and supplementary materials.

## Abbreviations

MIA: Maternal immune activation
LPS: Lipopolysaccharide
Poly I:C: Polyinosinic:polycytidylic acid
PPI: Prepulse inhibition
