# Supplementary material for: The prenatal challenge with lipopolysaccharide and polyinosinic:polycytidylic acid disrupts CX3CL1-CX3CR1 and CD200-CD200R signalling in the brains of male rat offspring: a link to schizophrenia-like behaviours
Source: J Neuroinflammation. 2020 Aug 23;17:247. doi: 10.1186/s12974-020-01923-0 (PMC7444338; doi:10.1186/s12974-020-01923-0)
Supplement: Supplementary file 1 — Additional file 1: Table S1. A list of genes (with corresponding catalogue numbers of TaqMan probes) examined in the hippocampi and the frontal cortices of male offspring at PND7 using qRT-PCR. B2m or Hprt were used as the reference genes. [file 12974_2020_1923_MOESM1_ESM.docx]

**Supplementary Table 1.**

| **Gene** | **Catalogue number** |
| --- | --- |
| *Cx3cl1* | Rn00593186_m1 |
| *Cx3cr1* | Rn00591798_m1 |
| *Cd200* | Rn01646320_m1 |
| *Cd200r* | Rn00576646_m1 |
| *MhcII* | Rn01424725_m1 |
| *Cd68* | Rn01495634_g1 |
| *Cd40* | Rn01423583_m1 |
| *iNos* | Rn00561646_m1 |
| *Il-1β* | Rn00580432_m1 |
| *Tnf-α* | Rn00562055_m1 |
| *Il-6* | Rn01410330_m1 |
| *Arg1* | Rn00691090_m1 |
| *Igf-1* | Rn00710306_m1 |
| *Tgf-β* | Rn00572010_m1 |
| *Il-4* | Rn01456866_m1 |
| *Il-10* | Rn01644839_m1 |
| *B2m* | Rn00560865_m1 |
| *Hprt* | Rn01527840_m1 |
