# Supplementary material for: The prenatal challenge with lipopolysaccharide and polyinosinic:polycytidylic acid disrupts CX3CL1-CX3CR1 and CD200-CD200R signalling in the brains of male rat offspring: a link to schizophrenia-like behaviours
Source: J Neuroinflammation. 2020 Aug 23;17:247. doi: 10.1186/s12974-020-01923-0 (PMC7444338; doi:10.1186/s12974-020-01923-0)
Supplement: Supplementary file 3 — Additional file 3: Figure S2. Immunohistofluorescent staining of CX3CL1-CX3CR1 (A, B) and CD200-CD200R (C, D) localization on neurons and microglial cells in the DG of the hippocampus of PND7 offspring after MIA induced by Poly I:C treatment. Representative confocal images showing colocalization of CX3CL1/CD200 (red) immunoreactivity with MAP2 (green)-positive neurons and CX3CR1/CD200R (red) immunoreactivity with IBA1 (green)-positive microglial cells. n = 2 in each group. Magnification: 40x for all images. Scale bar (10 μm) is located in the bottom right corner of each image. [file 12974_2020_1923_MOESM3_ESM.pdf]

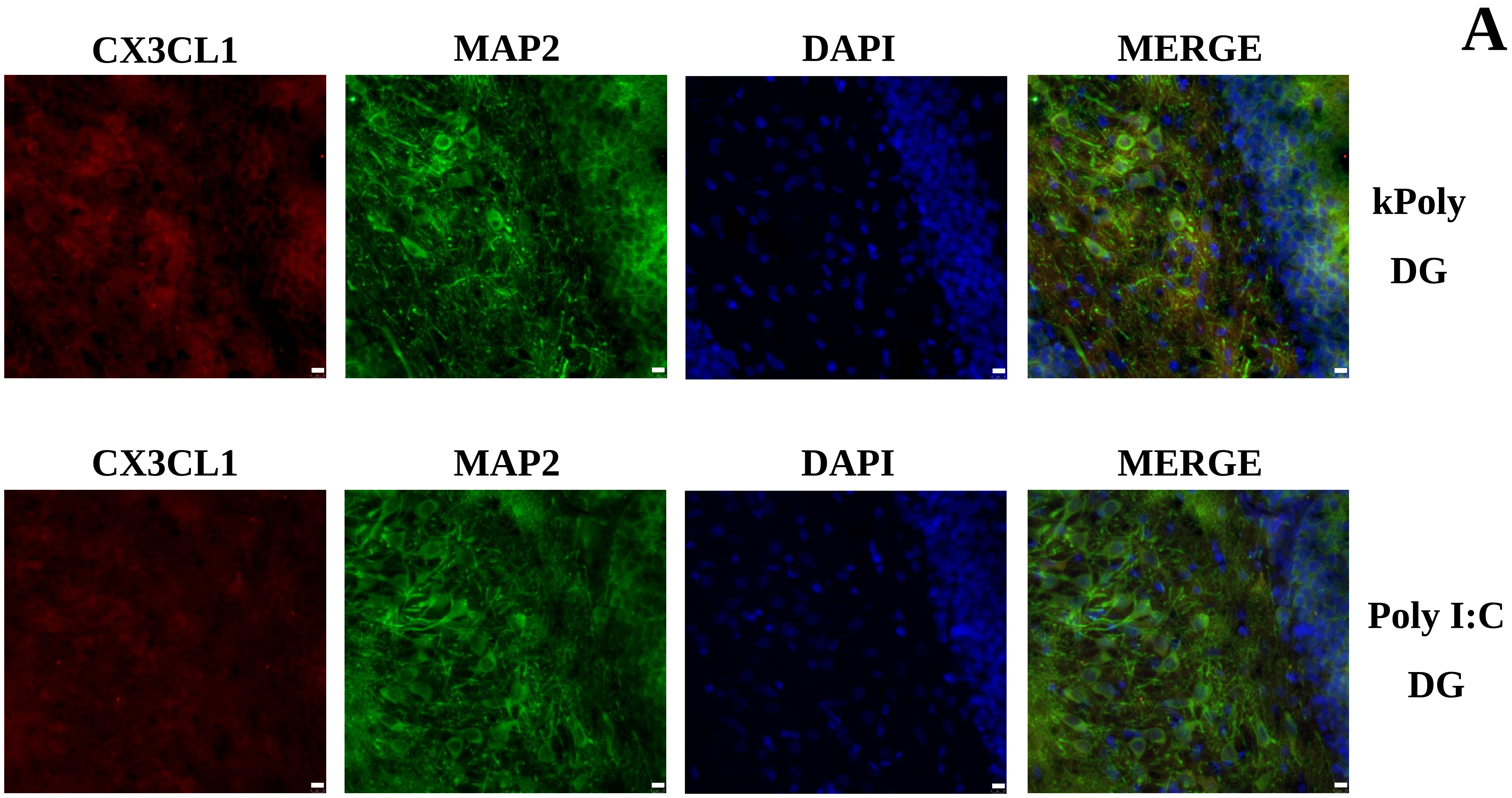
A
CX3CL1
MAP2
DAPI
MERGE
kPoly
DG
CX3CL1
MAP2
DAPI
MERGE
Poly I:C
DG

B

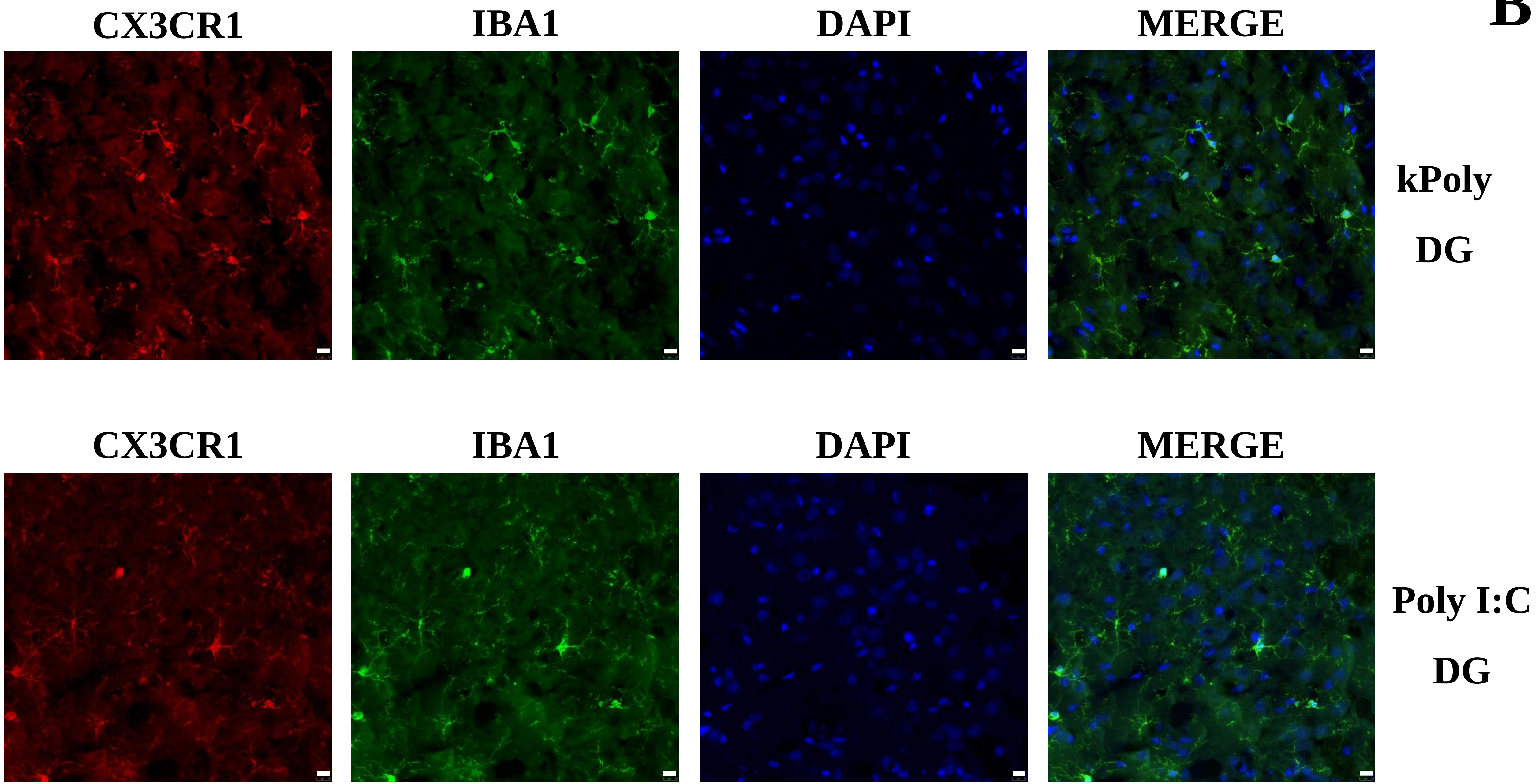
CX3CR1
IBA1
DAPI
MERGE
kPoly
DG
CX3CR1
IBA1
DAPI
MERGE
Poly I:C
DG

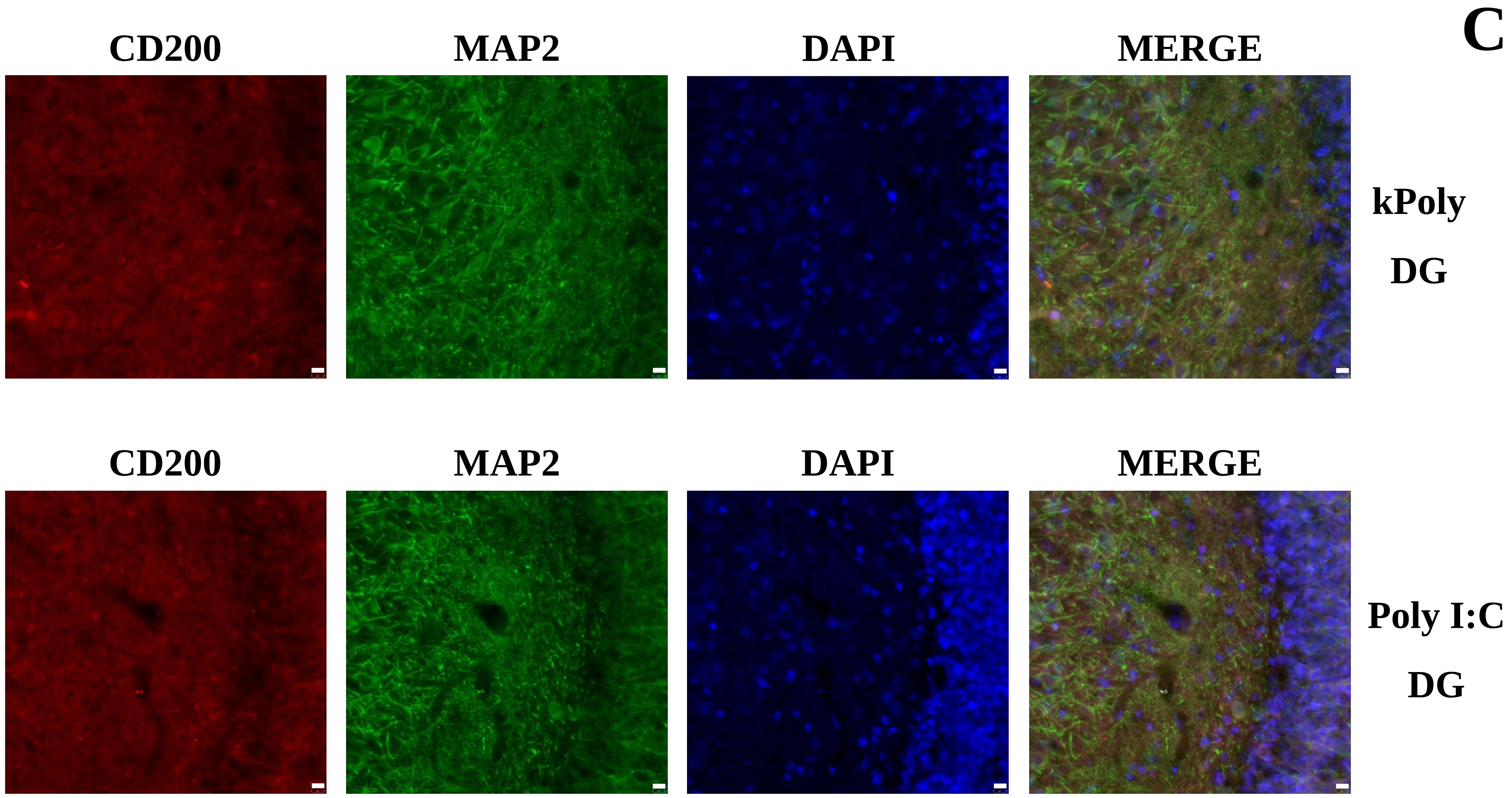
C
CD200
MAP2
DAPI
MERGE
kPoly
DG
CD200
MAP2
DAPI
MERGE
Poly I:C
DG

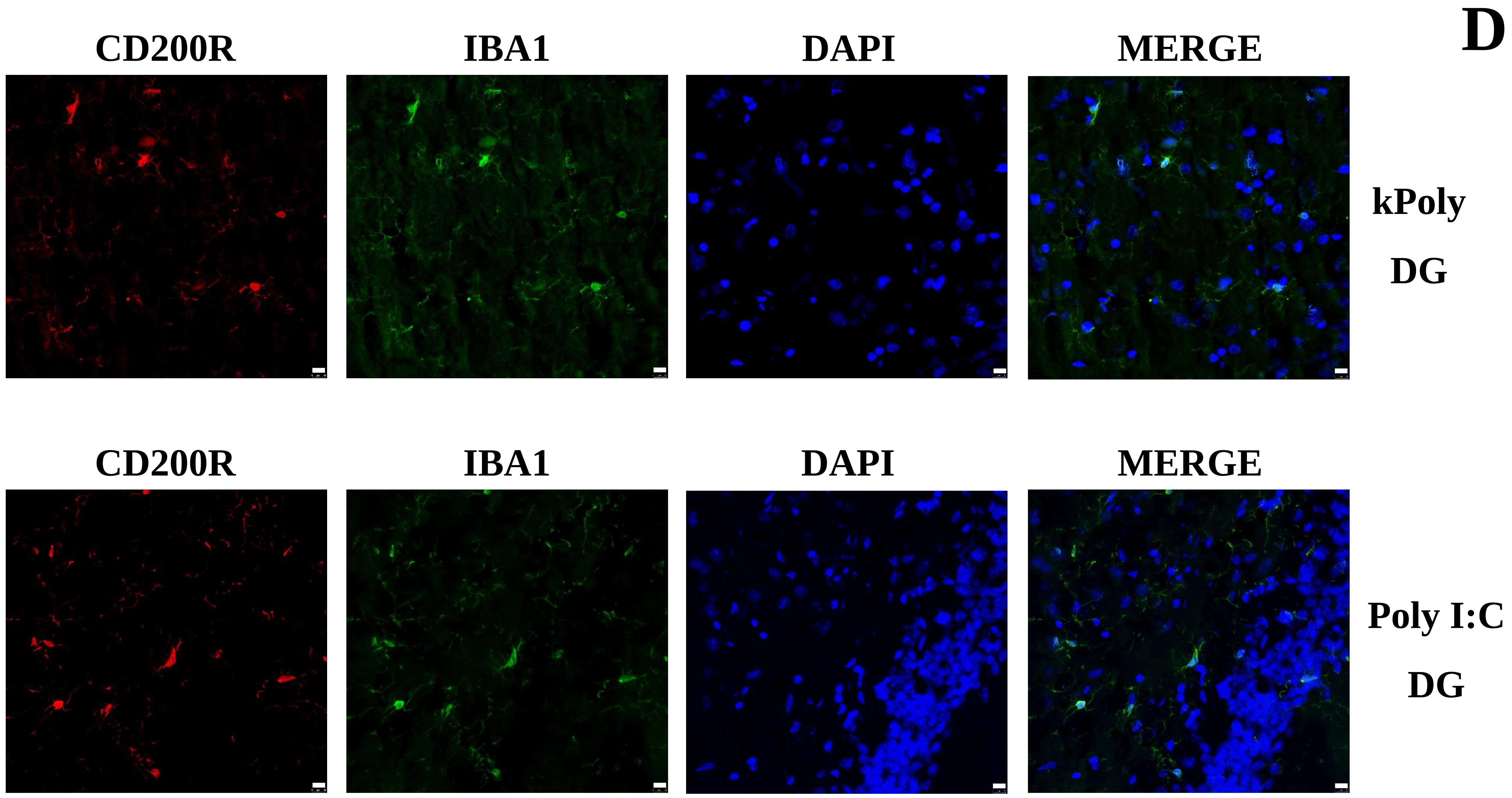
D
CD200R
IBA1
DAPI
MERGE
kPoly
DG
CD200R
IBA1
DAPI
MERGE
Poly I:C
DG
