# Supplementary material for: The prenatal challenge with lipopolysaccharide and polyinosinic:polycytidylic acid disrupts CX3CL1-CX3CR1 and CD200-CD200R signalling in the brains of male rat offspring: a link to schizophrenia-like behaviours
Source: J Neuroinflammation. 2020 Aug 23;17:247. doi: 10.1186/s12974-020-01923-0 (PMC7444338; doi:10.1186/s12974-020-01923-0)
Supplement: Supplementary file 6 — Additional file 6: Figure S5. Immunohistofluorescent staining of CX3CL1-CX3CR1 (A, B) and CD200-CD200R (C, D) localization on neurons and microglial cells in the CA3 field of the hippocampus of PND7 offspring after MIA induced by LPS treatment. Representative confocal images showing colocalization of CX3CL1/CD200 (red) immunoreactivity with MAP2 (green)-positive neurons and CX3CR1/CD200R (red) immunoreactivity with IBA1 (green)-positive microglial cells. n = 2 in each group. Magnification: 40x for all images. Scale bar (10 μm) is located in the bottom right corner of each image. [file 12974_2020_1923_MOESM6_ESM.pdf]

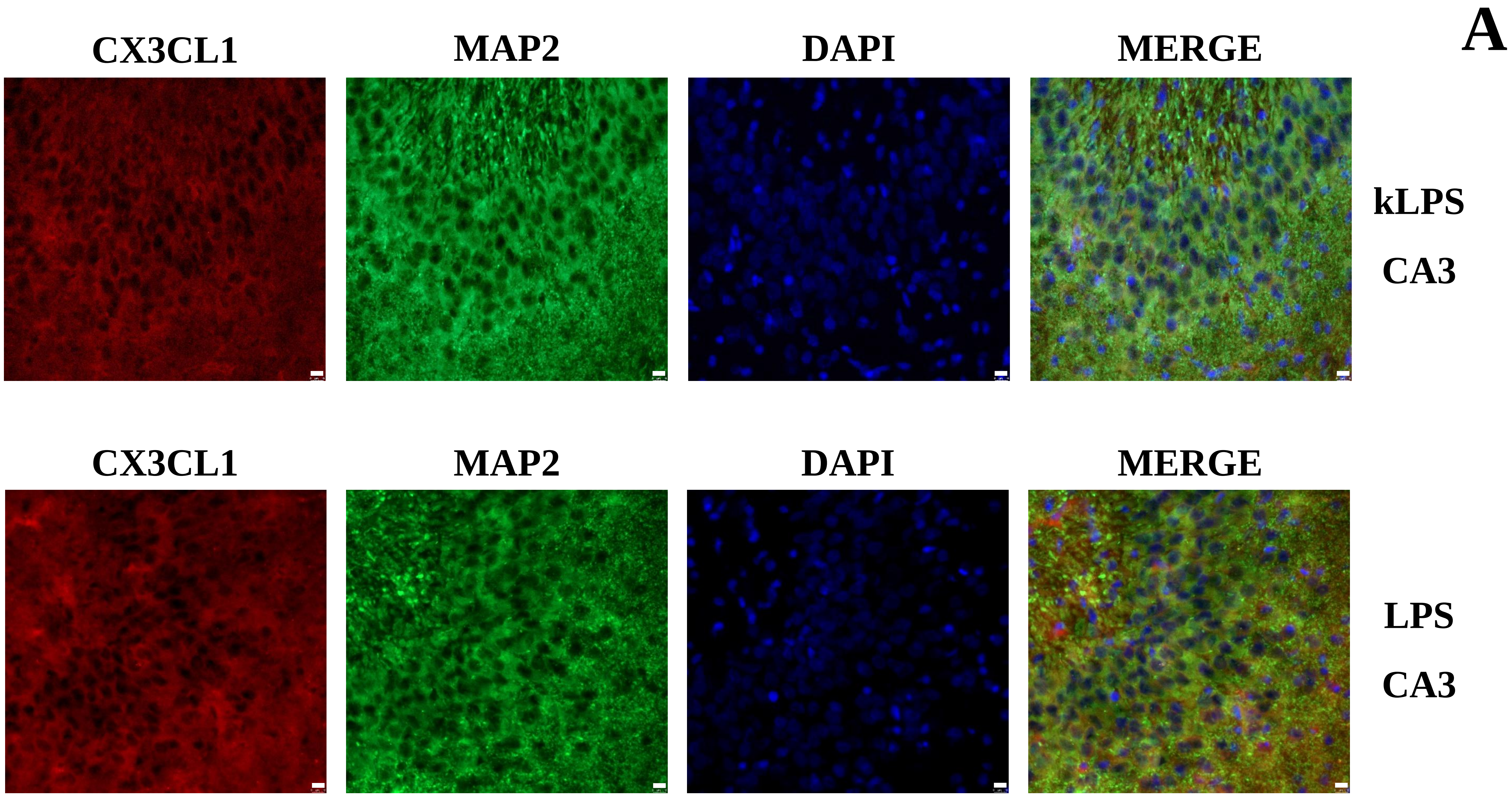
A
CX3CL1
MAP2
DAPI
MERGE
kLPS
CA3
CX3CL1
MAP2
DAPI
MERGE
LPS
CA3

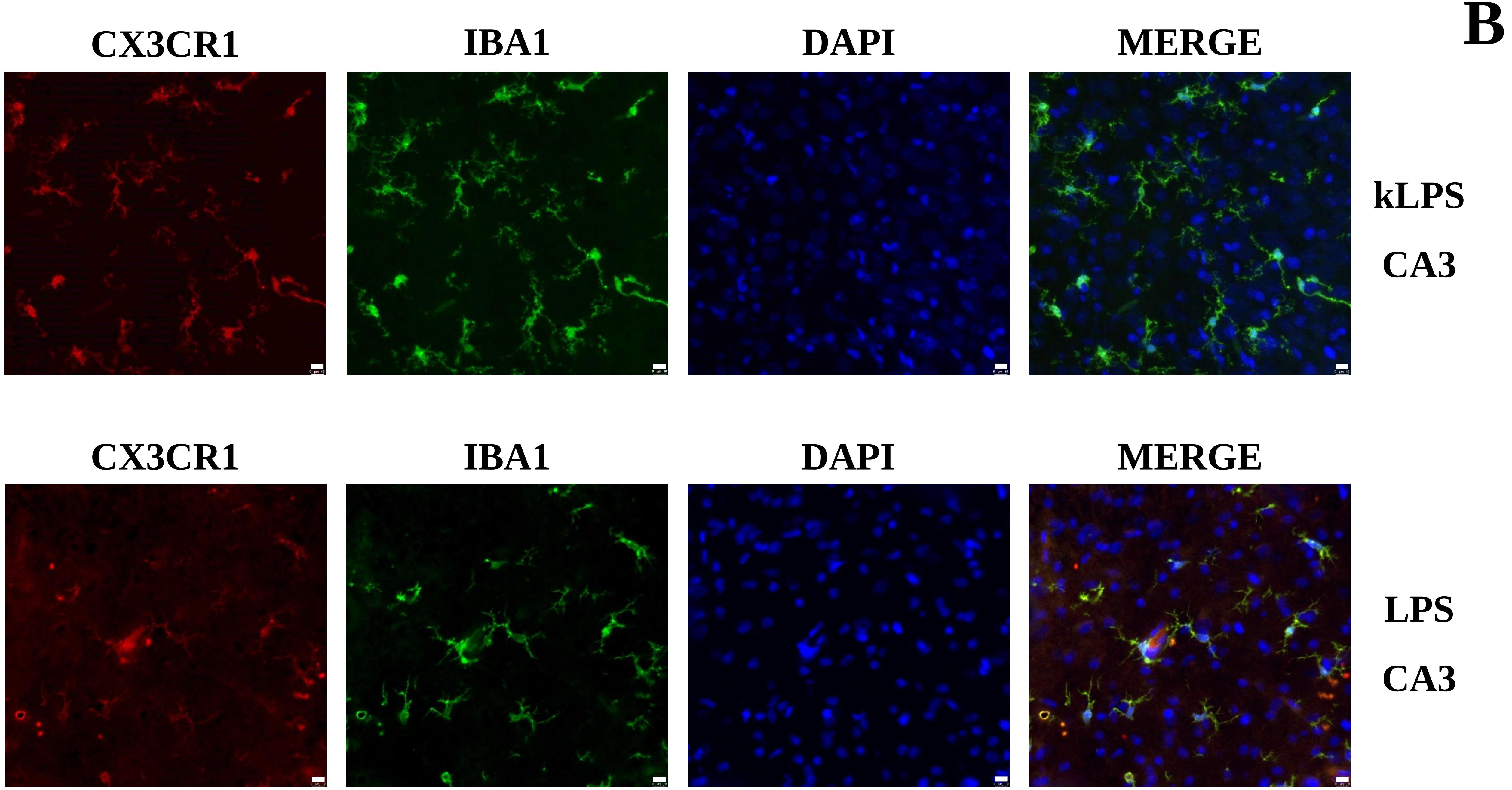
B
CX3CR1
IBA1
DAPI
MERGE
kLPS
CA3
CX3CR1
IBA1
DAPI
MERGE
LPS
CA3

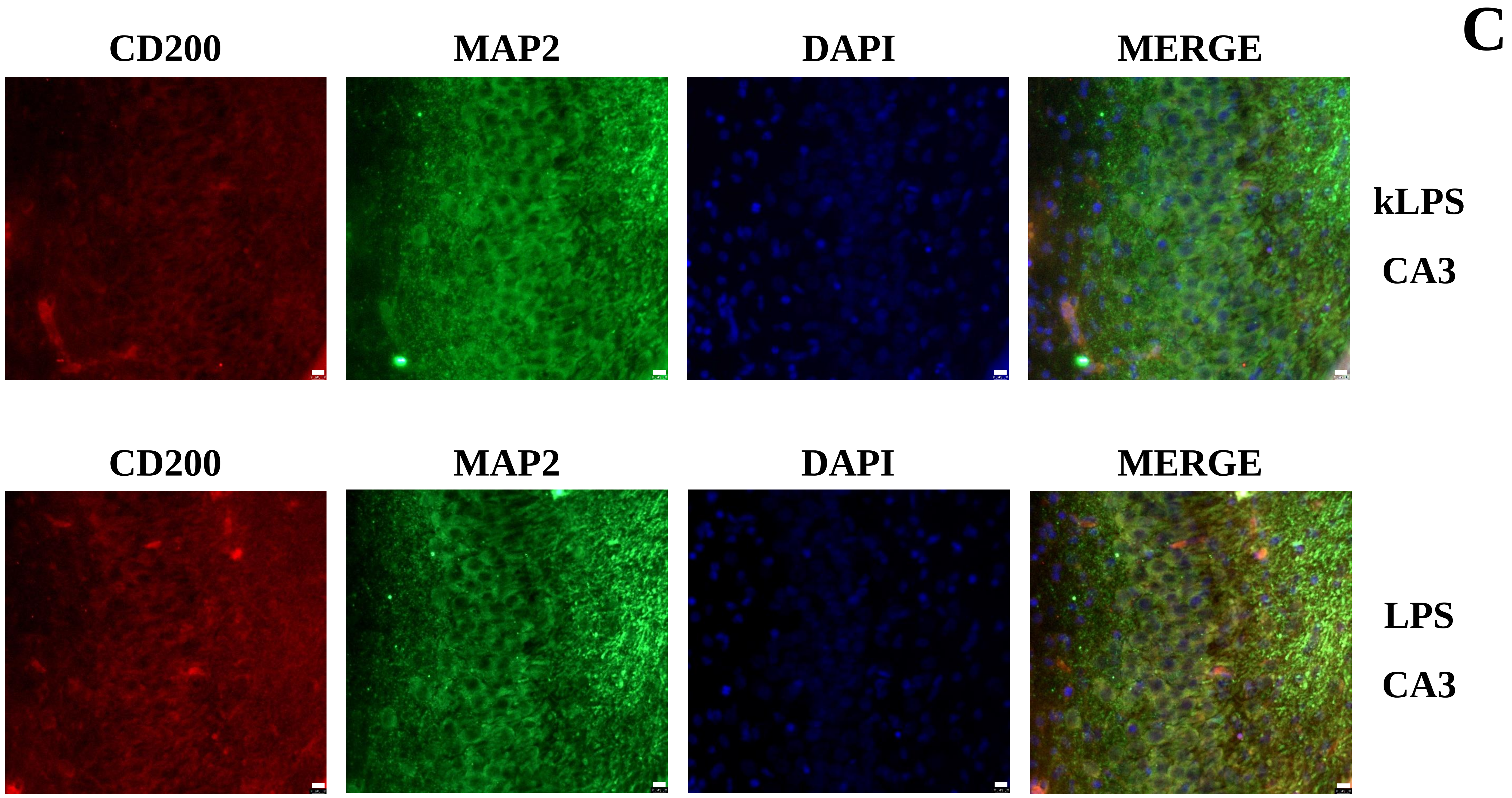
C
CD200
MAP2
DAPI
MERGE
kLPS
CA3
CD200
MAP2
DAPI
MERGE
LPS
CA3

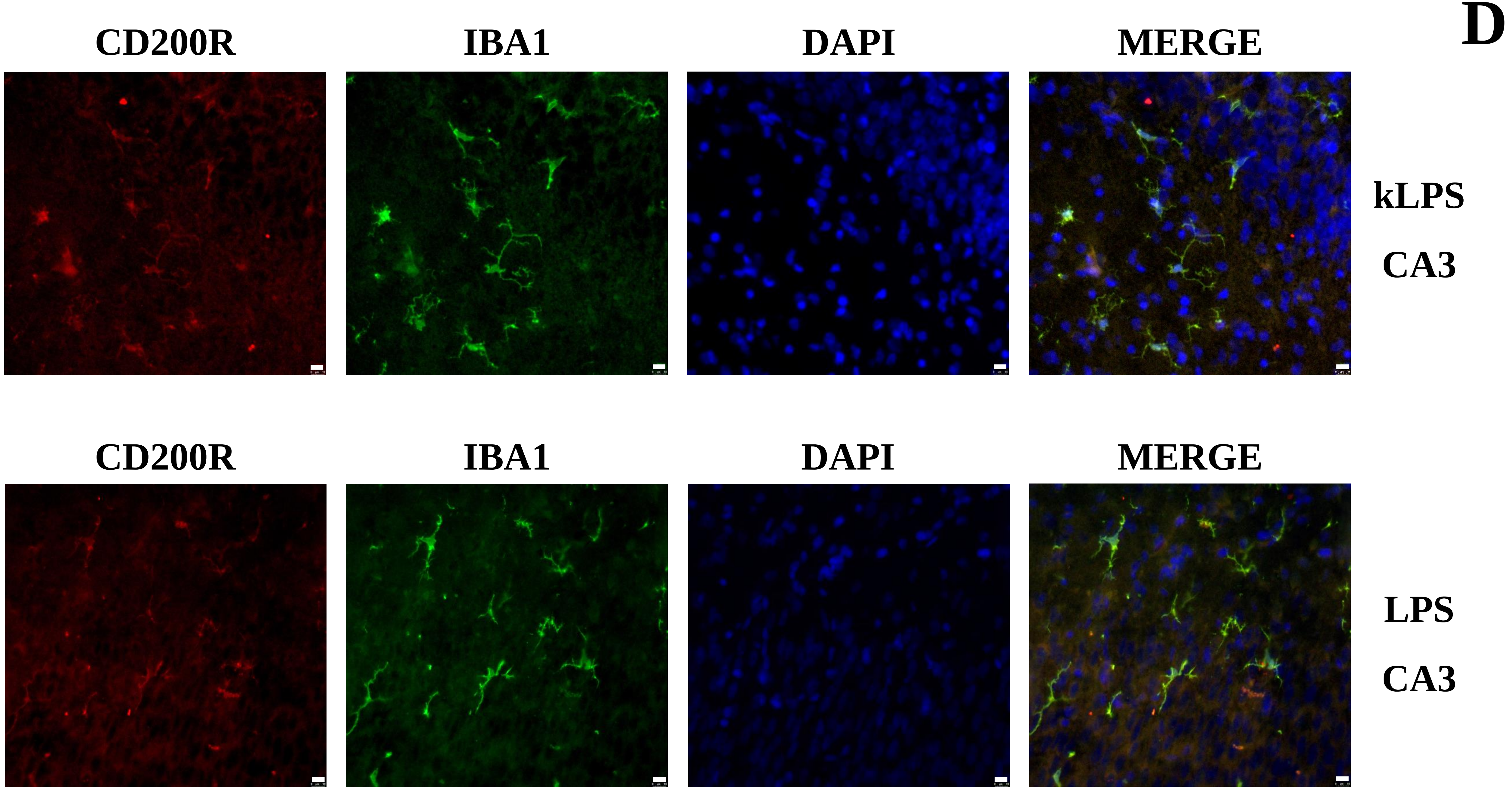
D
CD200R
IBA1
DAPI
MERGE
kLPS
CA3
CD200R
IBA1
DAPI
MERGE
LPS
CA3
